# Mesenchymal stem cells over-expressing *cxcl12* enhance the radioresistance of the small intestine

**DOI:** 10.1038/s41419-017-0222-1

**Published:** 2018-02-05

**Authors:** Pengyu Chang, Boyin Zhang, Lihong Shao, Wei Song, Weiyan Shi, Libo Wang, Tiankai Xu, Dong Li, Xiuzhu Gao, Yaqin Qu, Lihua Dong, Jin Wang

**Affiliations:** 1 State Key Laboratory of Electroanalytical Chemistry, Chinese Academy of Sciences, 130022 Changchun, China; 2 Department of Radiation Oncology, First Bethune Hospital of Jilin University, 130021 Changchun, China; 3 Department of Orthopedics Surgery, China-Japan Union Hospital of Jilin University, 130033 Changchun, China; 4 Department of Oncology, First Bethune Hospital of Jilin University, 130021 Changchun, China; 5 Department of Immunology, College of Basic Medical Sciences, Jilin University, 130021 Changchun, China; 6 Jilin Province Key Laboratory of Infectious Diseases, Laboratory of Molecular Virology, 130061 Changchun, China; 7 Department of Hepatology, First Bethune Hospital of Jilin University, Jilin University, 130021 Changchun, China; 8 Department of Chemistry and Physics, State University of New York at Stony Brook, New York, NY 11794-3400 USA

## Abstract

The chemokine C–X–C motif chemokine 12 (CXCL12) greatly impacts various biological processes in mammals, including cell survival, growth and migration. Mesenchymal stem cells (MSCs) are promising tools for carrying foreign genes to treat radiation-induced injuries in the intestinal epithelium. In this study, human adipose-derived MSCs were constructed to over-express the mouse *cxcl12* gene to treat such injuries. In vitro, because of the high levels of mouse CXCL12 in conditioned medium produced by mouse *cxcl12* gene-modified cells, phosphorylation of Akt at Ser473 and Erk1/2 at Thr202/Thr204 was increased within crypt cells of irradiated organoids compared with unmodified controls. Moreover, intracellular stabilization of β-catenin was achieved after treatment of mouse *cxcl12* gene-modified cells with conditioned medium. As a result, survival of crypt cells was maintained and their proliferation was promoted. When delivering mouse *cxcl12* gene-modified cells into irradiated BALB/c nude mice, mice were rescued despite the clearance of cells from the host within 1 week. Irradiated mice that received mouse *cxcl12* gene-modified MSCs exhibited reduced serum levels of interleukin-1α (IL-1α) and IL-6 as well as elevated levels of CXCL12. Additionally, epithelial recovery from radiation stress was accelerated compared with the irradiated-alone controls. Moreover, mouse *cxcl12* gene-modified MSCs were superior to unmodified cells at strengthening host repair responses to radiation stress as well as presenting increased serum CXCL12 levels and decreased serum IL-1α levels. Furthermore, the number of crypt cells that were positive for phosphorylated Akt at Ser473 and phosphorylated Erk1/2 at Thr202/Thr204 increased following treatment with mouse *cxcl12* gene-modified MSCs. Thus, *cxcl12* gene-modified MSCs confer radioresistance to the intestinal epithelium.

## Introduction

In healthy individuals, the intestinal epithelium constitutes a barrier for defence against aggressive luminal microbes^1^. However, several foreign stresses, such as ionizing irradiation (IR), forcefully impair this barrier and ultimately lead to microbial translocation, resulting in gastrointestinal dysfunction or even systematic infections^2^. Therefore, intestinal epithelial integrity is critical for human health.

De-epithelialization, microvascular destruction and inflammation are the main lesions of irradiated intestine^3^. Mesenchymal stem cells (MSCs) are potent tools for managing these lesions^4^. Several studies have revealed the excellent performance of MSCs in promoting epithelial regeneration, facilitating angiogenesis and reducing inflammation^5^. Recently, MSCs have been widely used in gene therapy for radiation-induced intestinal injury (RIII)^5^, because MSCs are capable of homing to injured sites based on their expression of chemokine receptors, such as CXCR4^6^. Moreover, over-expression of *cxcr4* gene by MSCs will further increase their homing efficacy and improve their ability to repair injured tissues^6-8^. During their migrations, stromal-derived factor-1α, also known as C–X–C motif chemokine 12 (CXCL12), plays a pivotal role^9^.

CXCL12 is capable of controlling cell survival, growth and migration during tissue/organ development^10^. The receptors of CXCL12 are CXCR4 and CXCR7. Among diverse cell types, CXCR4 and CXCR7 are expressed uniquely or in combination^11^. They form homodimers independently or heterodimers with each other to affect the biological processes^11^. For example, Akt and Erk can be activated when CXCL12 interacts with a CXCR4 homodimer, with a CXCR7 homodimer or with a CXCR4-CXCR7 heterodimer, respectively^11,12^. Nevertheless, cell migration event is largely attributed to the CXCL12–CXCR4 axis^12^. The CXCL12–CXCR7 axis is capable of promoting cell adhesion^12^. Alternatively, the CXCL12–CXCR7 axis inhibits the migration of cardiac stem cells by activating Akt^12^. Conversely, the CXCL12–CXCR4 axis maintained the migratory properties of cardiac stem cells^13^, implicating different roles of CXCR4 and CXCR7 in regulating cell migration.

In the small intestine, the epithelium represents a rapidly renewing tissue. Herein, canonical Wnt and MAPK/Erk signalling pathways are responsible for promoting the proliferation of intestinal stem/progenitor cells^14^. Additionally, activation of PI3K/Akt is critical for protecting intestinal crypts against radiation-induced death^15^. Accordingly, CXCR4 is expressed by epithelial cells^16^, and CXCL12 can be detected in intestinal tissue^9^. Upon binding, several biological effects should be triggered to assist epithelial homeostasis. A recent study reported that CXCL12 enables colorectal cancer stem cells to initiate transcription of *Cd44 v6* through activating the canonical Wnt^17^. Moreover, a previous study demonstrated that the CXCL12–CXCR4 axis preferentially activates PI3K/Akt and MAPK/Erk for repairing myocardial ischaemia/reperfusion injuries^18^. All these data support the therapeutic potential of CXCL12 in tissue injury.

MSCs are cellular sources of CXCL12^19^. Moreover, MSCs are ideal carriers of foreign *cxcl12* genes. In this study, by comparison with hAd-MSCs infected by empty lentiviral plasmid (termed ‘Lv-MSCs’ below), hAd-MSCs over-expressing the mouse *cxcl12* gene (termed ‘Lv-*cxcl12*-MSCs’ below) were used to evaluate their potential for repairing epithelial injury within irradiated intestine both in vitro and in vivo. Then, the mechanisms by which Lv-*cxcl12*-MSCs repaired epithelial injury were explored. The present results confirmed the therapeutic effects of Lv-*cxcl12*-MSCs on epithelial injuries within irradiated intestine.

## Results

### The cytokine profile of MSC-CM

**Fig. 1:**
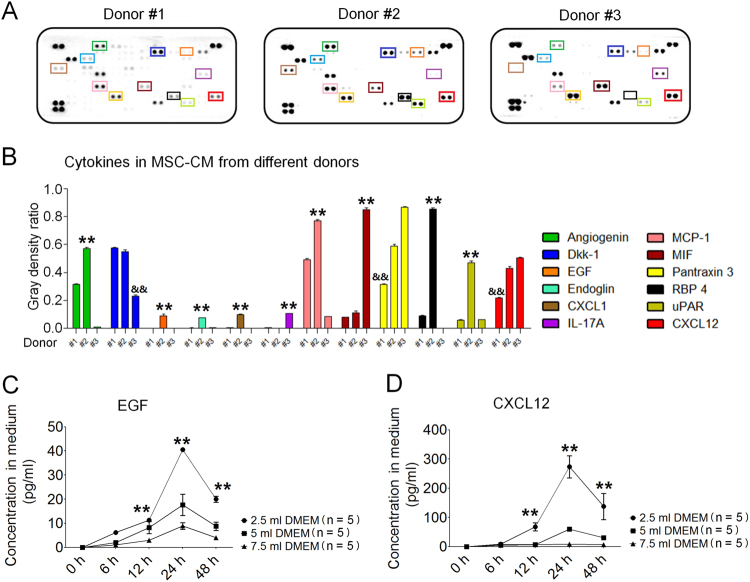
Cytokine profile of hAd-MSC. **a** Cytokine array for MSC-CM from three donors. Each cytokine was detected in duplicate. In each array, blots in the upper-left, upper-right and bottom-left represent positive controls. Target cytokines are indicated using square frames with different colours. Their names are listed in **b**. **b** Grey density analyses indicating the levels of cytokines in MSC-CM using Image Pro Plus 6.0 software. The mean value (*M*) of the grey density of three positive controls was calculated, and the grey density for each point of a target cytokine was divided by *M*, and this ratio was calculated in duplicate. The ratios were used to compare differences in the cytokine levels among MSC-CM from Donor #1, Donor #2 and Donor #3. Two-way ANOVA analysis was performed. **P ≤ 0.001: significant increase versus other two groups; ^&&^ ≤ 0.001: significant decrease versus the other two groups. **c** Human EGF levels in MSC-CM. Each time point consisted of five independent measurements (*n* = 5). Data represent the mean ± S.D. **P ≤ 0.001: significant increase (2.5 ml of DMEM group versus other two groups, one-way ANOVA). **d** Human CXCL12 levels in MSC-CM. Each time point consisted of five independent measurements (*n* = 5). Data represent the mean ± S.D. **P ≤ 0.001: significant increase (2.5 ml of DMEM group versus other two groups, one-way ANOVA)

MSCs are adult stem cells that can secrete various cytokines^4^. To select a superior cell line, we first compared the cytokine profiles of hAd-MSCs from three different donors. Passage 3 hAd-MSCs were cultured in serum-free Dulbecco's modified Eagle's medium (DMEM) for 24 h. Thereafter, a cytokine array analysis was performed using MSC-conditioned medium (MSC-CM). Here, we showed that MSC-CM from Donor #2 contained higher levels of cytokines, including angiogenin, endoglin, Dkk-1, EGF, RBP4 and CXCL12, but lower levels of interleukin-17A (IL-17A) than MSC-CM from the other two donors (Fig. 1a, b). Consequently, hAd-MSCs from Donor #2 were selected for further use.

The cytokines secreted by hAd-MSCs, including EGF^20^ and CXCL12^17^, were separately reported to be capable of activating MAPK/Erk among intestinal stem/progenitor cells^20^ and activating Wnt within colorectal cancer stem cells^17^. Next, we treated hAd-MSCs with different volumes of DMEM and collected MSC-CM at different time points to determine the concentrations of these cytokines. Herein, EGF and CXCL12 exhibited peak levels in MSC-CM that had been pre-conditioned for 24 h. Simultaneously, the highest concentrations of EGF and CXCL12 were detected in the 2.5 ml DMEM group (Fig. 1c, d).

### Lv-*cxcl12*-MSC-CM and crypt cell survival within irradiated organoids

**Fig. 2:**
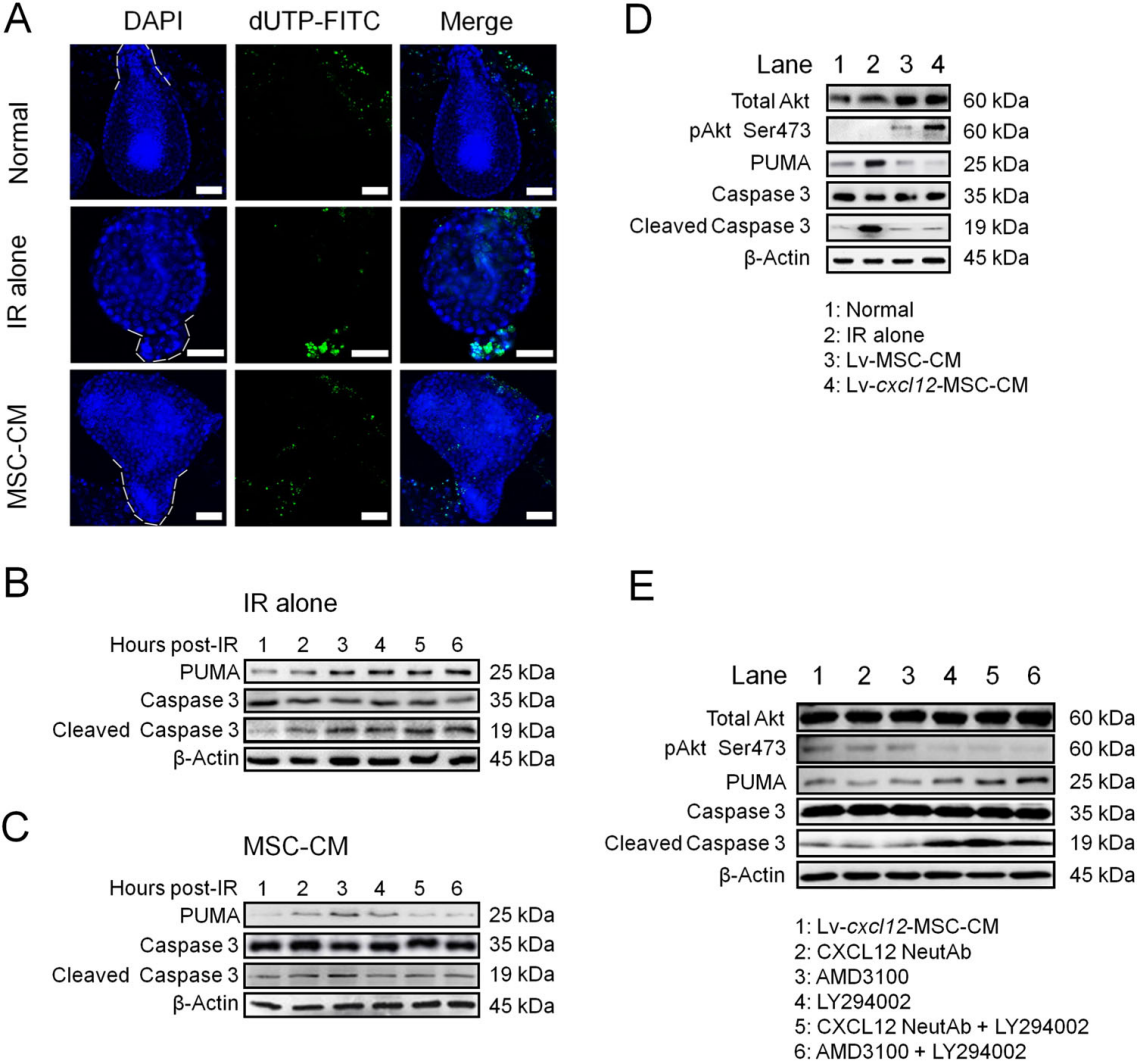
Cell apoptosis and Lv-*cxcl12*-MSC-CM. **a** In situ apoptosis detection within organoids using the terminal deoxynucleotidyl transferase dUTP nick-end labelling (TUNEL) assay at 6 h post IR. DAPI: nuclei; FITC: dUTP. Crypt domains were displayed using white dotted lines. Magnification (upper panel): 200×, scale bar: 100 μm; magnification (middle panel): 400×, scale bar: 100 μm; magnification (lower panel): 200×, scale bar: 100 μm. **b** Cell apoptosis within organoids during a period of 6 h post IR. Western blotting for PUMA caspase-3 and cleaved caspase-3 was performed. β-Actin was used as an internal control. The number represents the hour post IR. This experiment was carried out three times (*n* = 3) with similar results. **c** Cell apoptosis within irradiated organoids during a period of 6 h after treatment with MSC-CM. Western blotting for PUMA, caspase-3 and cleaved caspase-3 was performed. β-Actin was used as an internal control. The number represents the hour post IR. This experiment was carried out three times (*n* = 3) with similar results. **d** Activation of Akt and cell survival analysis within irradiated organoid cells after treatment with Lv-MSC-CM and Lv-*cxcl12*-MSC-CM for 6 h. Protein levels of total Akt, phosphorylated Akt at Ser473 (pAkt Ser473) PUMA, caspase-3 and cleaved caspase-3 were tested by western blotting. β-Actin was used as an internal control. Three independent measurements were performed (*n* = 3) with similar results. **e** Relationship between CXCL12–CXCR4 and Akt phosphorylation at Ser473. Lv-*cxcl12*-MSC-CM and Lv-*cxcl12*-MSC-CM containing 1 μg/ml of CXCL12 neutralizing antibody or 10 μM of AMD3100, 10 μM of LY294002, or 1 μg/ml of CXCL12 neutralizing antibody plus 10 μM of LY294002, or 10 μM of AMD3100 plus 10 μM of LY294002 were used to treat irradiated organoids for 6 h. Thereafter, protein levels of total Akt, pAkt Ser473, PUMA, caspase-3 and cleaved caspase-3 were evaluated by western blotting. β-Actin was used as an internal control. Three independent measurements were performed (*n* = 3) with similar results

Cell apoptosis, the main cause of intestinal crypt death, peaked at approximately 3 to 6 h after radiation^21^. Herein, PUMA (p53 up-regulated modulator of apoptosis), the BH3-only protein p53 up-regulated modulator of apoptosis, was reported to be capable of increasing the radiosensitivity of intestinal progenitor cells through inducing caspase activation^22^. Thus, the anti-apoptotic effect of MSC-CM on irradiated organoids was investigated. As shown in Fig. 2a, IR-induced apoptotic cells were restricted to the crypt domain, and cell apoptosis within this domain was remarkably inhibited after treatment with MSC-CM. In the IR-alone group, the levels of PUMA and cleaved caspase-3 gradually increased and peaked at 6 h post IR (Fig. 2b). In contrast, after treatment with MSC-CM, they exhibited the highest levels only at 3 h post IR (Fig. 2c) and then rapidly declined (Fig. 2c), indicating the onset time of MSC-CM against cell apoptosis began at ~4 h post IR. Moreover, at 6 h post IR, flow-cytometric analysis revealed that the amounts of apoptotic cells both at early and late stages in MSC-CM group were significantly lower than those in IR-alone group (Supplementary Figure S1), indicating that MSC-CM protected against radiation-induced cell death.

Next, we compared the differences in Lv-MSCs and Lv-*cxcl12*-MSCs in protecting irradiated organoid cells. Prior to this process, the distributions of CXCR4^+^ and CXCR7^+^ cells were mapped within the intestinal epithelium. In the small intestine, most of the CXCR4^+^ cells were located within crypts (Supplementary Figure S2A), whereas no CXCR7^+^ cells were observed in the epithelium (data not shown). In addition, the level of mouse CXCL12 in Lv-*cxcl12*-MSC-CM (in 2.5 ml of serum-free DMEM for 24 h) was 62.6 ± 5.4 ng/ml (Supplementary Figure S3). In contrast, mouse CXCL12 was not detected in Lv-MSC-CM. Nevertheless, organoids treated with either Lv-MSC-CM or Lv-*cxcl12*-MSC-CM were more resistant to IR than organoids that received IR alone as well as presented decreased levels of PUMA and cleaved caspase-3 and increased levels of Akt phosphorylated at Ser473 (Fig. 2d). As mentioned above, activation of PI3K/Akt was associated with increased radioresistance of intestinal crypts because PUMA was suppressed upon activation of this signalling pathway^15^. In accordance with this finding, the present results showed lower PUMA levels in the Lv-*cxcl12*-MSC-CM group than in the Lv-MSC-CM group. Moreover, the level of phosphorylated Akt at Ser473 was further increased in response to treatment with Lv-*cxcl12*-MSC-CM (Fig. 2d), thus demonstrating that mouse CXCL12 could facilitate Akt activation.

Next, we tested whether mouse CXCL12 protected irradiated organoid cells against apoptosis via PI3K/Akt signalling pathway. A positive relationship between phosphorylated Akt at Ser473 and the mouse CXCL12 concentration in CM was identified, and CXCR4^+^ cells were mostly restrained within crypt domain. Based on these results, the blocking assay was performed. However, we failed to observe that either CXCL12 neutralizing antibody or AMD3100 (an antagonist of CXCR4) altered the intracellular levels of phosphorylated Akt at Ser473, PUMA and cleaved caspase-3, which were not in equivalent to the levels following treatment with LY294002 (the inhibitor of PI3K) (Fig. 2e). These findings suggested that CXCL12 was involved in activating Akt but was not the unique factor for Akt activation.

### Lv-*cxcl12*-MSC-CM and crypt cell proliferation within irradiated organoids

**Fig. 3:**
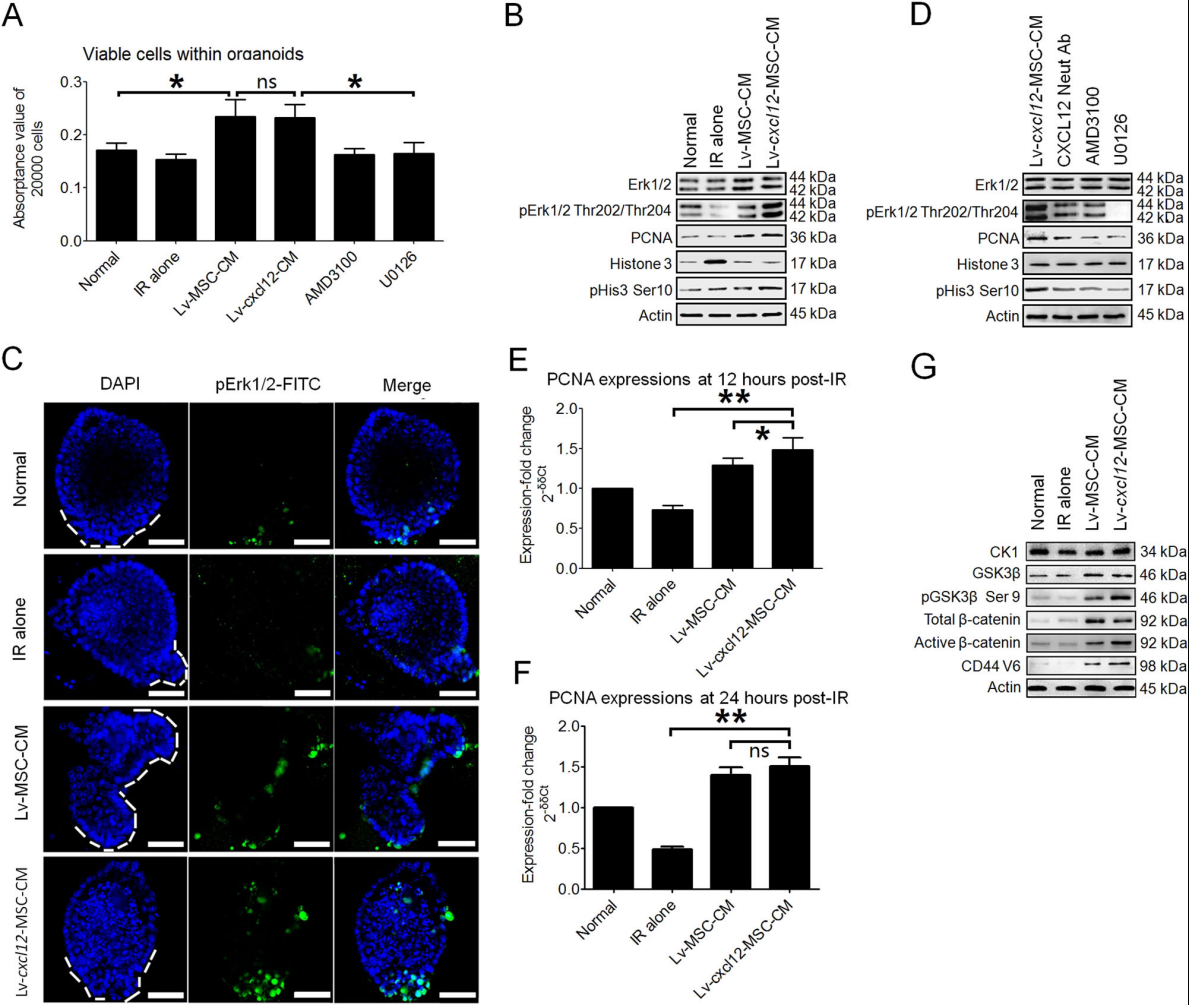
Cell proliferation and Lv-*cxcl12*-MSC-CM. **a** Cell viability analysis using PrestoBlue reagent. Irradiated organoids were seeded onto a 96-well plate. Lv-MSC-CM, Lv-*cxcl12*-MSC-CM and Lv-*cxcl12*-MSC-CM containing 10 μM of AMD3100 and Lv-*cxcl12*-MSC-CM containing 10 μM of U0126 were added separately to the wells. Normal organoids served as healthy controls. Each group contained eight wells (*n* = 8). Six hours later, the organoids were dissociated into single cells. Each 20,000 cells were harvested from each well and stained with 1× PrestoBlue solution for 10 min. Thereafter, the absorbance values were calculated. Data are the mean ± S.D. *P ≤ 0.05: significant increase (Lv-MSC-CM group versus the Normal and IR-alone groups, one-way ANOVA; Lv-*cxcl12*-MSC-CM group versus AMD3100 and U0126 groups, one-way ANOVA analysis) (Lv-MSC-CM group versus Lv-*cxcl12*-MSC-CM group). **b** Activation of Erk1/2 and cell proliferation within irradiated organoids after treatment with Lv-MSC-CM and Lv-*cxcl12*-MSC-CM for 6 h. Protein levels of Erk1/2, phosphorylated Erk1/2 (pErk1/2) at Thr202/Thr204, PCNA, histone 3 and phosphorylated histone 3 at Ser10 (pHis3 Ser10) were tested by western blotting. β-Actin was used as an internal control. Three independent measurements were performed (*n* = 3) with similar results. **c** Phosphorylation of Erk1/2 (pErk1/2) in irradiated crypt cells after treatment with Lv-MSC-CM and Lv-*cxcl12*-MSC-CM for 6 h. Crypt domains are displayed using white dotted lines. Confocal images were captured. DAPI: nuclei; FITC: pErk1/2. Magnification: 400×; scale bar: 100 μm. **e** Relationship between CXCL12–CXCR4 and Erk1/2 phosphorylation at Thr202/Thr204. Lv-*cxcl12*-MSC-CM and Lv-*cxcl12*-MSC-CM containing 1 μg/ml of CXCL12 neutralizing antibody, 10 μM of AMD3100, or 10 μM of U0126 were used to treat irradiated organoids for 6 h. Thereafter, protein levels of Erk1/2, pErk1/2, PCNA, histone 3 and pHis3 Ser10 were tested by western blotting. β-Actin was used as an internal control. Three independent measurements were performed (*n* = 3) with similar results. **f** *PCNA* expression in irradiated organoid cells 12 h after Lv-MSC-CM and Lv-*cxcl12*-MSC-CM treatment. Real-time PCR was performed. *GAPDH* was used as an internal control. The fold-increase was normalized to the Normal group according to the 2^−δδCt^ algorithm. Data in each group represent the mean ± S.D. of six independent measurements (*n* = 6). **P ≤ 0.001: significant increase (Lv-*cxcl12*-MSC-CM group versus IR-alone group, unpaired *t* test); *P ≤ 0.05 (Lv-*cxcl12*-MSC-CM group versus Lv-MSC-CM group, unpaired *t* test). NS: no significance. **g** *PCNA* expression in irradiated organoid cells 24 h after Lv-MSC-CM and Lv-*cxcl12*-MSC-CM treatment. Real-time PCR was performed. *GAPDH* was used as an internal control. The fold-increase was normalized to the Normal group according to the 2−^δδCt^ algorithm. Data in each group represent the mean ± S.D. of six independent measurements (*n* = 6). **P ≤ 0.001: significant increase (Lv-*cxcl12*-MSC-CM group versus IR-alone group, unpaired *t* test); NS: no significant difference (Lv-*cxcl12*-MSC-CM group versus Lv-MSC-CM group, unpaired *t* test). **h** β-Catenin stabilization in irradiated organoid cells after treatment with Lv-MSC-CM and Lv-*cxcl12*-MSC-CM for 6 h. Protein levels of CK-1, GSK3β, phosphorylated GSK3β at Ser9 (pGSK3β Ser9), total β-catenin and active β-catenin were tested by western blotting. β-Actin was used as an internal control. Three independent measurements were performed (*n* = 3) with similar results

CXCL12 has been reported to be capable of activating the MAPK/Erk signalling pathway^18^, which accounted for approximately 50% of mitosis among intestinal stem/progenitor cells^20^. Mitosis would maintain the amount of viable cells within organoids. Thus, the irradiated organoids were treated with CM from Lv-MSCs and Lv-*cxcl12*-MSCs for 6 h. Then, cell viabilities within irradiated organoids were compared among groups. In contrast to the Normal and IR-alone groups, the cell viabilities within irradiated organoids were significantly enhanced after treatment with CM (Fig. 3a). In this context, we examined whether MAPK/Erk was activated following treatment with CM. The levels of phosphorylated Erk1/2 at Thr202/Thr204 in Lv-MSC-CM group were maintained similar to those in healthy organoids, in contrast to their obvious loss in the IR-alone group. Moreover, these protein levels were further increased in response to treatment with Lv-*cxcl12*-MSC-CM (Fig. 3b), demonstrating that mouse CXCL12 could promote phosphorylation of Erk1/2 at Thr202/Thr204. As a result, the levels of proteins indicating DNA replication, such as proliferating cell nuclear antigen (PCNA)^23^, and cell mitosis, such as phosphorylated histone 3 at Ser10^24^, were further increased (Fig. 3b). And the phosphorylated Erk1/2-positive cells were constrained within crypts (Fig. 3c). Next, using a blocking assay, we examined the specificity of mouse CXCL12 for activating Erk1/2. Relevant results showed that the levels of PCNA and phosphorylated histone 3 at Ser10 were decreased when irradiated organoids were treated with CXCL12 neutralizing antibodies AMD3100 and U0126 (a MEK inhibitor), respectively (Fig. 3d). Likewise, cell viabilities within irradiated organoids were significantly reduced after the addition of AMD3100 or U0126 to Lv-*cxcl12*-MSC-CM (Fig. 3a). Taken together, these results indicated that activation of Erk1/2 by CXCL12–CXCR4 axis allowed Lv-*cxcl12*-MSC-CM to promote proliferation among irradiated crypt cells.

Next, we assessed cell proliferation within organoids at 12 and 24 h post IR because Dkk-1, an antagonist of Wnt^25^, was detected in MSC-CM (Supplementary Figure S4), which potentially inhibited the proliferation of intestinal stem/progenitor cells^25^. Intriguingly, both Lv-MSC-CM and Lv-*cxcl12*-MSC-CM still enabled significantly high expression of *PCNA* gene compared with IR-alone group (Fig. 3e, f). Herein, up-regulated *PCNA* expressions after treatment with Lv-MSC-CM or with Lv-*cxcl12*-MSC-CM seemed to be attributed to increased phosphorylation of GSK3β at Ser9, which resulted in intracellular stabilization of β-catenin^26^. Moreover, in comparison to Lv-MSC-CM, Lv-*cxcl12*-MSC-CM enabled the level of phosphorylated GSK3β at Ser9 to further increase, leading to a higher level of active β-catenin. In this context, the level of CD44 V6 protein, a target of Wnt, was obviously increased (Fig. 3g). Similarly, a recent study confirmed that CXCL12 was capable of promoting initiation of the transcription of *Cd44 v6* in colorectal cancer stem cells by activating Wnt^17^.

### Lv-*cxcl12*-MSCs and prolonged survival of irradiated mice

**Fig. 4:**
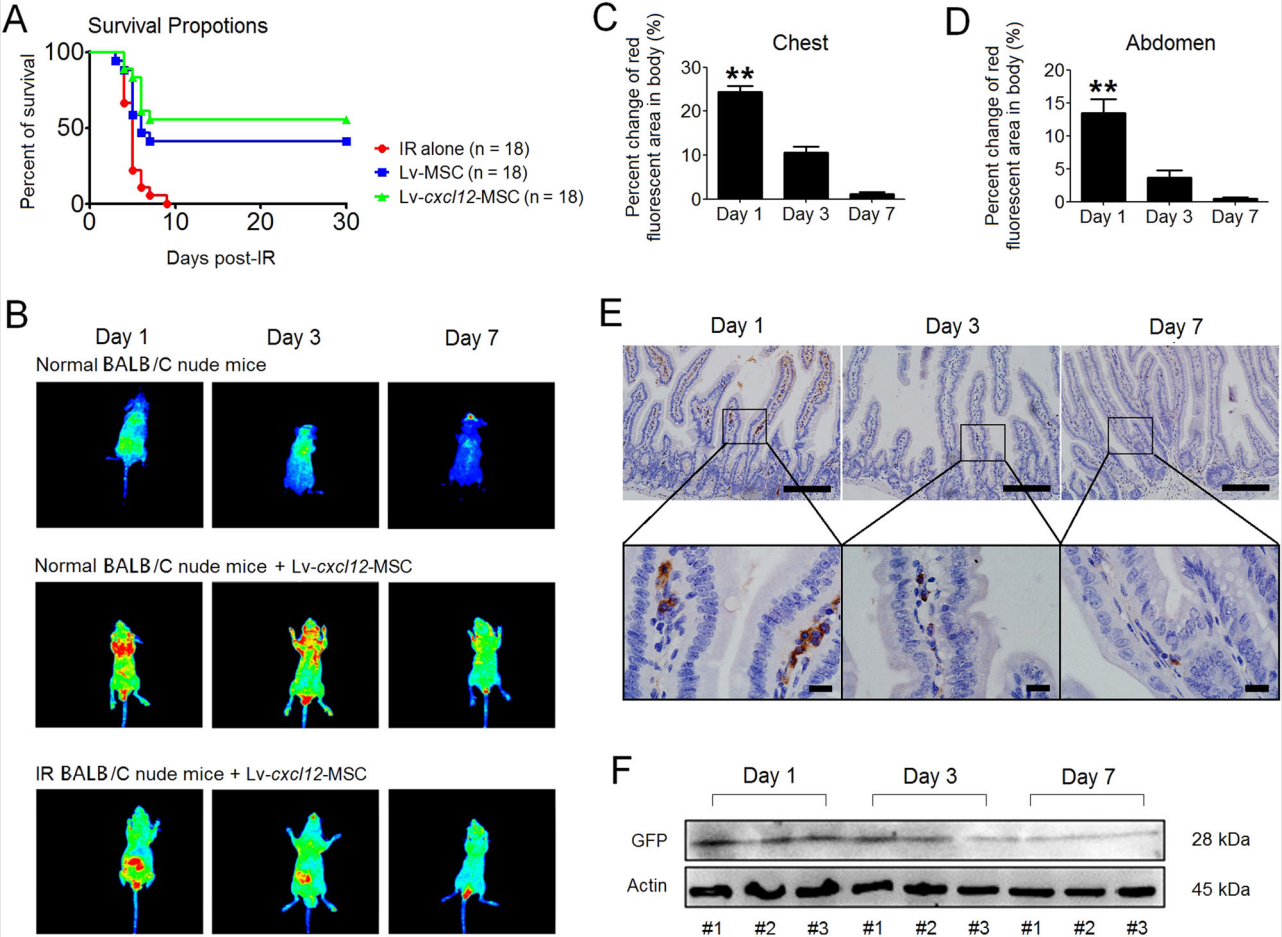
Delivery of Lv-cxcl12-MSCs and prolonged survival of irradiated mice. **a** Survival analysis of irradiated BALB/c nude mice using Kaplan–Meier methodology. Each group contained 18 mice (*n* = 18). **b** In vivo distribution of Lv-*cxcl12*-MSCs. The red field denotes higher GFP fluorescence signals than the green field. The blue field represents the background fluorescence. The distribution of Lv-*cxcl12*-MSCs in irradiated mice was traced at 1 day, 3 days and 7 days. The images in each group were captured from the same mouse. **c** The clearance of Lv-*cxcl12*-MSCs from healthy BALB/c mice. The red field in the chest was calculated using Image Pro Plus 6.0 software. Data represent the mean ± S.D. of three independent measurements (*n* = 3). **P ≤ 0.001: significant increase (day 1 group versus other groups, one-way ANOVA). **d** The clearance of Lv-*cxcl12*-MSCs from irradiated BALB/c mice. The red field in the abdomen was calculated using Image Pro Plus 6.0 software. Data represent the mean ± S.D. of three independent measurements (*n* = 3). **P ≤ 0.001: significant increase (day 1 group versus other groups, one-way ANOVA). (**e**) Alterations in the distributions of Lv-*cxcl12*-MSCs in the small intestine during a period of 7 days post IR. IHC staining for GFP. Upper panel: magnification: 200×, scale bar: 100 μm; lower panel: magnification: 1000×, scale bar: 20 μm. **f** Alterations in the amounts of Lv-*cxcl12*-MSCs in the small intestine during a period of 7 days post IR. Each three independent measurements (*n* = 3) were carried out by western blotting for GFP levels in irradiated intestine at 1 day, 3 days and 7 days post IR. β-Actin was used as an internal control

Our in vitro study showed that Lv-*cxcl12*-MSCs had advantages over Lv-MSCs in activating Akt and Erk1/2. In vivo, the entire abdomens of BALB/c nude mice were irradiated with a single dose of 10.5 Gy^27^. Thereafter, Lv-MSCs or Lv-*cxcl12*-MSCs were intraperitoneally injected into irradiated mice. Mice that only received IR served as controls. During a 30-day observation period, mice in the IR-alone group died within 9 days post IR (Fig. 4a). In contrast, Lv-MSCs and Lv-*cxcl12*-MSCs rescued 38.9 and 55.6% of the irradiated mice, respectively (Fig. 4a). Then, by tracing GFP^+^ cells (Supplementary Figure S3), we analysed the distribution of Lv-*cxcl12*-MSCs in vivo. At 1 day post-cell delivery, Lv-*cxcl12*-MSCs were mainly located in the chest of healthy mice (Fig. 4b). In contrast, cells were mainly distributed in the abdomen of irradiated mice (Fig. 4b). Thereafter, in both healthy and irradiated mice, the scope of GFP signal rapidly narrowed over the following days (Fig. 4b–d), indicating the clearance of Lv-*cxcl12*-MSCs from the hosts. The time-dependent reduction of cell number in irradiated small intestine was also confirmed by immunohistochemical (IHC) staining and by western blotting for GFP (Fig. 4e, f). Based on these results, we confirmed that delivery of Lv-*cxcl12*-MSCs could prolong the survival of irradiated mice, although cells were cleared from the hosts within 1 week.

### Lv-*cxcl12*-MSCs and accelerated epithelial recovery

**Fig. 5:**
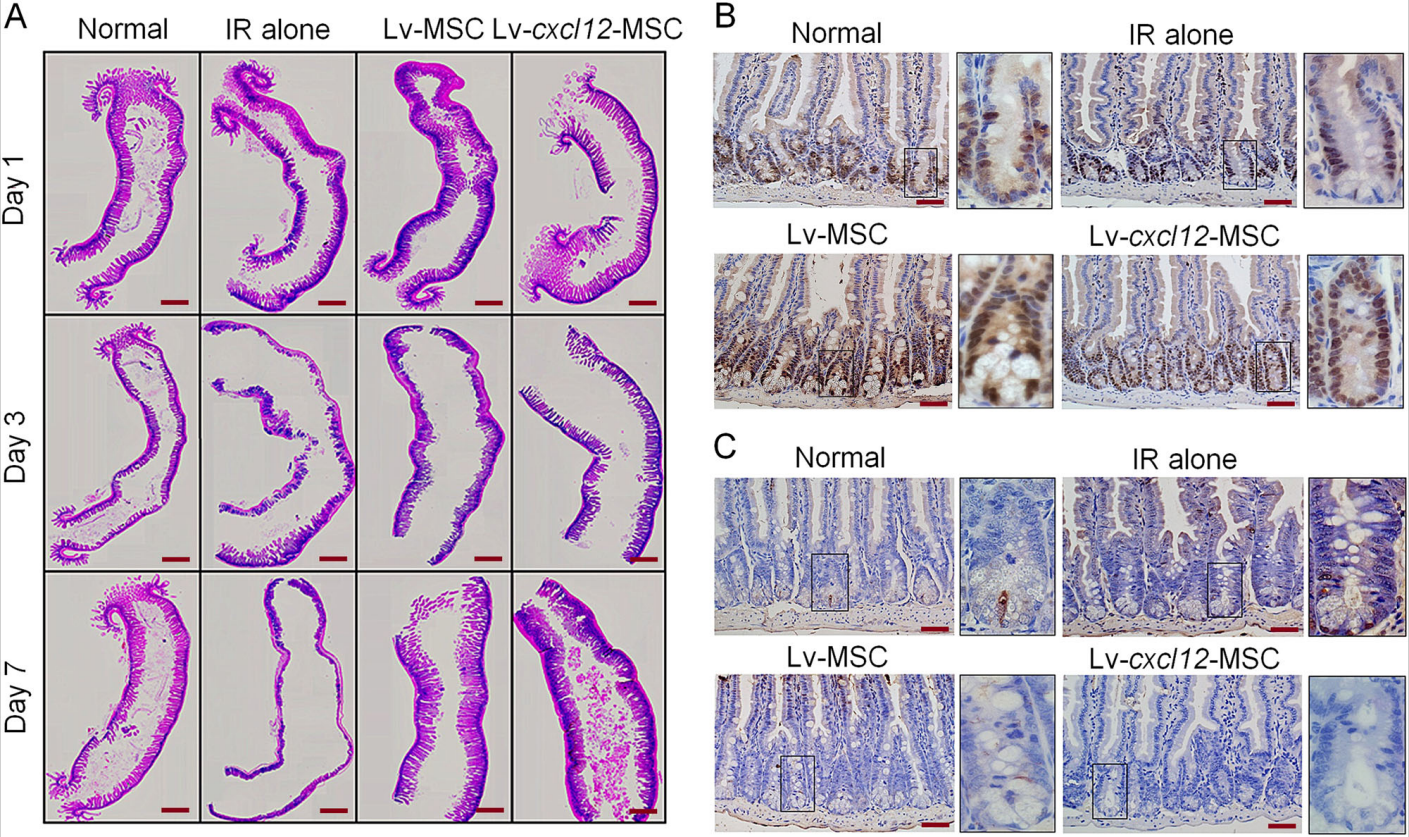
Delivery of Lv-*cxcl12*-MSCs and accelerated epithelial recovery. **a** Epithelial integrity analysis. H&E staining of the irradiated small intestine with a length of 1.5 cm was performed at 1 day, 3 days and 7 days post IR. The images were captured at 40× magnification. All images were merged into one image. Scale bar: 10 mm. **b** Cell proliferation analysis. IHC staining for PCNA was carried out at 3 days post IR. Representative images were captured for all groups. Magnification at 400×; scale bar: 50 μm. The images in black frames were captured at 1000× magnification. **c** Cell apoptosis analysis. TUNEL staining for in situ cell apoptosis was performed at 3 days post IR. Representative images were captured for all groups. Magnification: 400×; scale bar: 50 μm. The images in black frames were captured at 1000× magnification

Because Lv-*cxcl12*-MSCs rescued more irradiated BALB/c nude mice than Lv-MSCs, we next compared the histological differences in these mice after treatment with Lv-*cxcl12*-MSCs and Lv-MSCs. As shown in Fig. 5a, the epithelium of small intestine in IR-alone group was completely lost within 7 days post IR. In contrast, the intestinal epithelium at irradiated site was preserved in the Lv-MSC group. Moreover, in the Lv-*cxcl12*-MSCs group, radiation-induced de-epithelialization was not that severe as in the Lv-MSC group. This was because irradiated crypts from mice that received Lv-*cxcl12*-MSCs possessed more proliferative cells and fewer apoptotic cells than the Lv-MSC group (Fig. 5b, c), indicating that Lv-*cxcl12*-MSCs accelerated epithelial recovery from radiation stress.

### Lv-*cxcl12*-MSCs and strengthened host repair responses to radiation stress

**Fig. 6:**
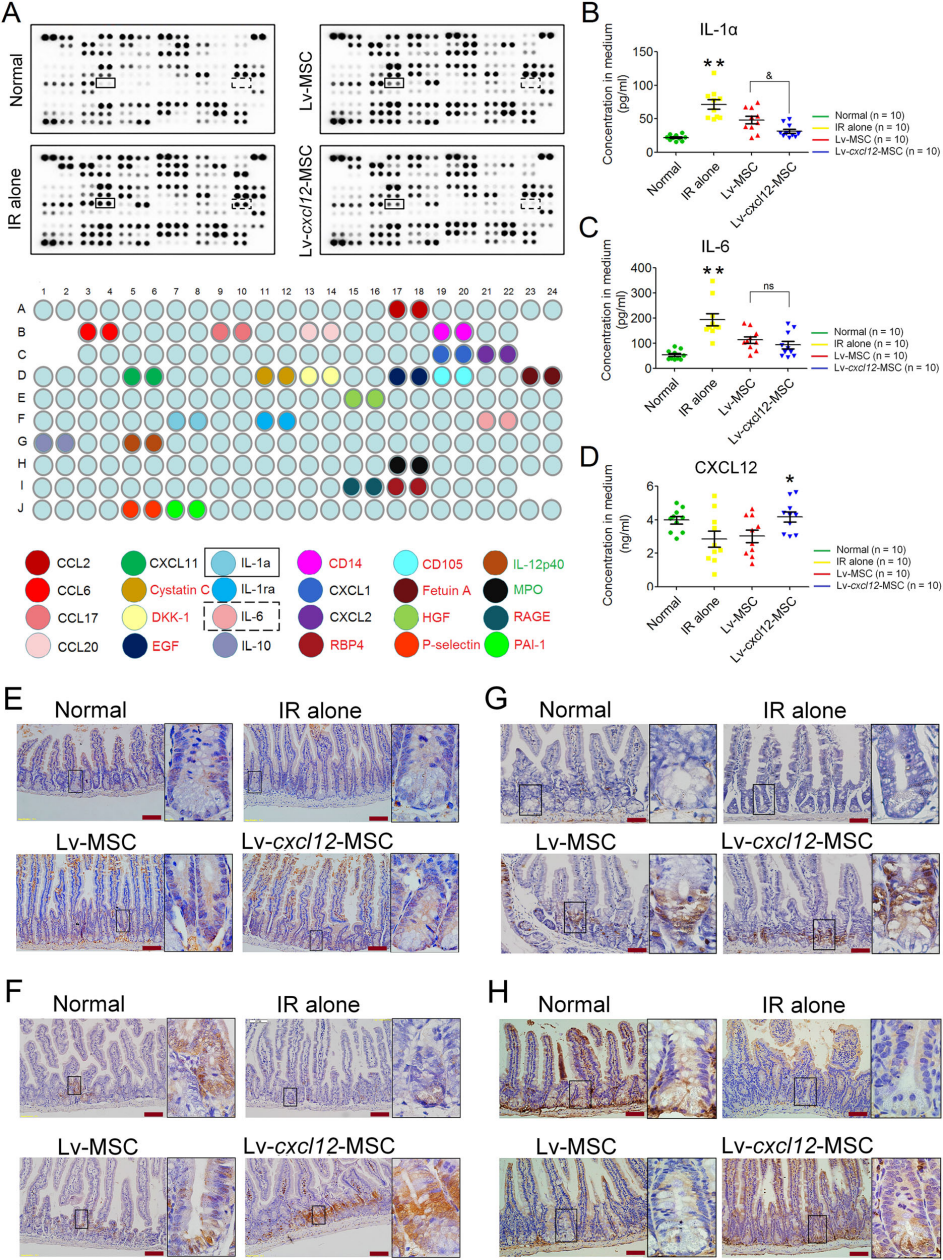
Delivery of Lv-*cxcl12*-MSCs and strengthened host repair responses. **a**) Serum cytokine array at 3 days post IR. The upper image shows immunoblots. The lower image shows the distribution of cytokines in the blots. Black font: cytokines with high serum levels only in the IR-alone group; red font: IR-induced cytokines with high serum levels; green font: IR-induced cytokines with low serum levels. Black box: IL-1α. Black box with a dashed line: IL-6. **b** Serum levels of IL-1α. ELISA for testing mouse IL-1α was performed at 3 days post IR. Each group consisted of 10 independent samples (*n* = 10). Data represent the mean ± S.D. ^**^P ≤ 0.001: significant increase (IR-alone group versus other three groups, one-way ANOVA); ^&^*P ≤ *0.05: significant decrease (Lv-*cxcl12*-MSC group versus Lv-MSC group, unpaired *t* test). **c** Serum levels of IL-6. IL-6 was measured at 3 days post IR by ELISA. Each group contained 10 independent samples (*n* = 10). Data represent the mean ± S.D. ^**^P ≤ 0.001: significant increase (IR-alone group versus other three groups, one-way ANOVA); NS: no significance (Lv-*cxcl12*-MSC group versus Lv-MSC group, unpaired *t* test). **d** Serum levels of CXCL12. CXCL12 was measured at 3 days post IR by ELISA. Each group contained 10 independent samples (*n* = 10). Data represent the mean ± S.D. ^*^P ≤ 0.05: significant increase (Lv-*cxcl12*-MSC group versus other three groups, one-way ANOVA). **e** Distribution of CXCR4^+^ cells within the irradiated intestinal epithelium. IHC staining for CXCR4 was performed at 3 days post IR. Representative images are shown for all groups. Magnification: 200×; scale bar: 100 μm. The images in black frames were captured at 1000× magnification. **f** Distribution of pERK1/2^+^ cells within the irradiated intestinal epithelium. IHC staining for pERK1/2 was performed at 3 days post IR. Representative images are shown for all groups. Magnification: 200×; scale bar: 100 μm. The images in black frames were captured at 1000× magnification. **g** Distribution of pAkt Ser473^+^ cells within the irradiated intestinal epithelium. IHC staining for pAkt Ser473 was performed at 3 days post IR. Representative images are shown for all groups. Magnification: 200×; scale bar: 100 μm. The images in black frames were captured at 1000× magnification. **h** Distribution of Lgr5+ cells within the irradiated intestinal epithelium. IHC staining for Lgr5 was performed at 3 days post IR. Representative images are shown for all groups. Magnification: 200×; scale bar: 100 μm. The images in black frames were captured at 1000× magnification

Due to the accelerated recovery of epithelium after treatment with Lv-*cxcl12*-MSCs, we then analysed host responses to IR stress. As shown in Fig. 6a, several chemotactic ligands, including CCL2, CCL6, CCL17, CCL20 and CXCL11, along with inflammatory cytokines, including IL-1α, IL-1ra, IL-6 and IL-10, were produced at high levels in irradiated controls at 3 days post IR. In contrast, the delivery of both Lv-MSCs and Lv-*cxcl12*-MSCs significantly decreased their levels in serum (Fig. 6a). Additionally, regardless of cell delivery, the serum levels of several cytokines were increased after abdominal radiation, such as EGF, HGF, CD105, RBP4 and Dkk-1, whereas the levels of IL-12p40 and MPO were decreased compared with those in healthy controls (Fig. 6a), reflecting intrinsic responses of the host to radiation stress.

The serum cytokine array confirmed the decreased levels of IL-1α and IL-6 at 3 days after the delivery of Lv-MSCs and Lv-*cxcl12*-MSCs (Fig. 6a). By quantifying their serum concentrations, IL-1α and IL-6 levels were significantly reduced in both the Lv-MSC and Lv-*cxcl12*-MSC groups compared with the IR-alone group (*P* < 0.0001) (Fig. 6b, c). Moreover, the serum IL-1α level was significantly reduced in the Lv-*cxcl12*-MSC group compared with the Lv-MSC group (*P* = 0.0217) (Fig. 6b), whereas there were no differences in IL-6 levels between groups (Fig. 6c). In addition, the serum CXCL12 levels were decreased in both the IR-alone group and Lv-MSC group compared with the healthy controls, and the serum level of CXCL12 was elevated after delivering Lv-*cxcl12*-MSCs in comparison to the other groups (*P = *0.0229) (Fig. 6d). Altogether, Lv-*cxcl12*-MSCs showed improved host repair responses to IR stress to a greater extent than Lv-MSCs.

Locally, moderate numbers of Lv-*cxcl12*-MSCs were preserved within the villus stroma for 1 to 3 days post IR (Fig. 4e). Herein, GFP staining was useful to monitor the expression of the mouse *cxcl12* gene by MSCs within injured sites because its coding sequence was inserted before the *IRES-eGFP* locus (Supplementary Figure S3). Despite not determining the concentration of mouse CXCL12 within the irradiated intestine, more CXCR4^+^ cells were still located within crypt domains in the Lv-*cxcl12*-MSC group compared with the Lv-MSC group at 3 days post IR (Fig. 6e). Our in vitro study also confirmed that Lv-*cxcl12*-MSC-CM could further increase the protein level of CXCR4 compared with Lv-MSC-CM (Supplementary Fig. 2b). In this context, it should be beneficial to facilitate the activation of PI3K/Akt and MAPK/Erk among crypt cells upon CXCL12 intervention. In fact, the present results revealed that more crypt cells were positive for phosphorylated Akt at Ser473 and phosphorylated Erk1/2 at Thr202/204 after treatment with Lv-*cxcl12*-MSCs, as compared with Lv-MSCs (Fig. 6f, g), facilitating epithelial recovery from IR-induced damage. As a result, a large amount of Lgr5^+^ cells were identified within crypts (Fig. 6h), indicating that more intestinal stem/progenitor cells were preserved^28^. Overall, the delivery of Lv-*cxcl12*-MSCs was superior to Lv-MSCs in strengthening the host repair responses to radiation stress.

## Discussion

The present study revealed the therapeutic effects of Lv-*cxcl12*-MSCs on radiation-induced epithelial injury both* in* vitro and in vivo. Because CXCR4^+^ cells were fairly constrained within intestinal crypts, CXCL12 could impact the survival and growth among these CXCR4^+^ crypt cells.

In vitro, we confirmed the anti-apoptotic effects of MSC-CM on crypt cells within irradiated organoids. Moreover, mouse CXCL12 was capable of activating Akt because increased phosphorylation of Akt at Ser473 was positively correlated with elevated mouse CXCL12 levels in CM. As documented, PI3K-mediated phosphorylation at Ser473 of Akt conferred its full activation, which enhanced cell survival^29^. Thus, fewer apoptotic cells were observed in irradiated organoids after Lv-*cxcl12*-MSC-CM treatment, and low levels of PUMA and cleaved caspase-3 were detected. However, the blocking assay did not reveal the specific relationship between CXCL12 and the phosphorylation of Akt at Ser473, indicating that CXCL12 was not the unique factor for Akt activation. As we know, multiple factors can activate Akt^29^. For example, we found that MSC-CM contained EGF, a potent cytokine for Akt activation in the same way^30^. Besides, the CXCL12–CXCR4 axis was shown to regulate cell migration^10^. For maintaining the intestinal barrier integrity, previous data demonstrated that the CXCL12–CXCR4 axis stimulates epithelial migration in vitro^31,32^, a biological process mediated by PI3K and Erk1/2 MAP kinase^31^. Thus, when the progeny of intestinal stem cells migrate along the villus–crypt axis to mature, the CXCL12–CXCR4 axis can impact this process.

The proliferation among intestinal stem cells is an important biological event for epithelial homeostasis. Herein, MAPK/Erk and canonical Wnt were critical pathways regulating this process^20^. In this study, we found that Lv-*cxcl12-*MSC-CM could promote Erk1/2 phosphorylation at Thr202/Thr204 using the CXCL12–CXCR4 axis. In the Lv-MSC-CM group, phosphorylation of Erk1/2 was still maintained at levels similar to those in the Normal group. Herein, human EGF and CXCL12 were detected in MSC-CM. Thus, their contributions to Erk1/2 activation could not be excluded^10,33^. Canonical Wnt is regarded as the main driving force for proliferation among intestinal stem cells^20^. Herein, β-catenin is a very important factor for initiating the transcription of Wnt target genes, such as *Cd44 variant 6*^34^. In the absence of Wnt, β-catenin is phosphorylated by CK-1 at Ser45 and then by GSK3β at Ser33, Ser37 and Thr41^25^. Ultimately, β-catenin is degraded by ubiquitination^25^. In this study, although Dkk-1 was detected in MSC-CM, the expression of *PCNA* genes by organoid cells retained high levels after treatment with either Lv-MSC-CM or Lv-*cxcl12*-MSC-CM for up to 24 h post IR. Moreover, the higher levels of active β-catenin were achieved after treatment with Lv-*cxcl12*-MSC-CM and Lv-MSC-CM. Herein, intracellular stabilization of β-catenin has been reported to be associated with the Ser473-phosphorylated Akt molecule, which inactivates GSK3β by phosphorylating it at Ser9^26^. After treatment with Lv-*cxcl12*-MSC-CM, the levels of phosphorylated GSK3β at Ser9 and phosphorylated Akt at Ser473 were higher than those in the Lv-MSC-CM group. Consequently, the level of active β-catenin was further increased in the Lv-*cxcl12*-MSC-CM group.

In vitro, CXCL12 could account for the specific roles of Lv-*cxcl12*-MSCs in maintaining survival and promoting proliferation among irradiated crypt cells. However, such effects should not be replicated to illustrate the performance of Lv-*cxcl12*-MSCs in vivo. First, regardless of the use of irradiated mice or healthy controls, Lv-*cxcl12*-MSCs were cleared from the host after 1 week. Herein, xenograft rejection driven by effective T cells was reported to be a main approach for MSC clearance from the host. However, BALB/c nude mice were T cell deficient, which could not account for the MSC clearance. Nevertheless, transplantation of Lv-*cxcl12*-MSCs still revealed their therapeutic potentials, which were largely attributed to the strengthened repair responses of the host to radiation stress. For example, the delivery of Lv-*cxcl12*-MSCs decreased serum levels of IL-1α and IL-6 while increasing levels of CXCL12. Locally, obvious proliferation and a reduced extent of apoptosis were main biological events within irradiated crypts.

Moreover, Lv-*cxcl12*-MSCs showed advantages over Lv-MSCs in rescuing irradiated mice. For example, in addition to the lower serum IL-1α and higher CXCL12 than in the Lv-MSC group, the number of crypt cells that were positive for phosphorylated Akt at Ser473, phosphorylated Erk1/2 at Thr202/Thr204 and Lgr5 increased markedly after treatment with Lv-*cxcl12*-MSCs, as compared to Lv-MSCs. Herein, *Lgr5*, a target of Wnt, are expressed by intestinal stem/progenitor cells^28^. As reported previously, MSCs repaired intestinal injury by activating canonical Wnt, which maintained the protein levels of active β-catenin and Lgr5 in irradiated intestine^35^. In the present study, we also observed massive active β-catenin^+^ cells within irradiated intestinal crypts after delivery of Lv-*cxcl12*-MSCs (Supplementary Figure S5), indicating Lv-*cxcl12*-MSCs also activated canonical Wnt when repairing epithelial injury.

For clinical use of MSCs, one key issue is whether these cells promote tumour progression because excessive transcription of the *c-Myc* gene by active Wnt has been identified as a phenotype among some colorectal cancers^36^. Recently, MSC therapy was reported to be useful for relieving abdominal pain, stanching rectal bleeding and repairing fistulas among patients with prostate cancer who received an overdose of radiotherapy^37^. Moreover, previous data have suggested that down-regulated expression of *c-Myc* gene is one of the biological effects of hAd-MSCs on leukaemia cells^38^. But for Lv-*cxcl12*-MSCs, overproduction of CXCL12 would assist metastasis of CXCR4^+^ cancer cells, including colorectal cancer cells^39^. Nevertheless, we believe that the potential risks of Lv-*cxcl12*-MSCs can be theoretically quantified by identifying the critical elements such as genes or gene regulations responsible for that from the underlying gene regulatory networks by characterizing the Waddington landscapes and biological paths for normal and cancer developments as well as the cancer stem cells under various environmental conditions including epigenetics^40–49^. This will be the future work for us to explore.

In conclusion, Lv-*cxcl12*-MSCs confer radioresistance to the intestinal epithelium and that Akt and Erk1/2 activation is involved in this process.

## Materials and Methods

### MSC preparation

Subcutaneous fat was harvested from two healthy donors, namely, Donor #1 and Donor #2, as described below. The donors approved the use of their fat samples for isolating MSCs. The details of the procedures used to isolate MSCs have been previously reported^50^. In addition, another line of primary hAd-MSCs was purchased from ScienCell Research Laboratories (San Diego, CA, USA), namely, Donor #3 described below. Complete medium used to culture hAd-MSCs consisted of 95% DMEM-low glucose (LG)/F12 (ScienCell) and 5% FBS (ScienCell) supplemented with 1× mesangial cell growth supplement (ScienCell) and a 1× penicillin/streptomycin solution (ScienCell). Cells were cultured at 37 °C in a humidified atmosphere with 5% CO_2_. The medium was replaced every 2 to 3 days, and passaging was performed when cells reached approximately 80% confluence. Passage 3 hAd-MSCs were collected for further use.

### MSC phenotype

Passage 3 hAd-MSCs from Donor #2 were collected for phenotypic analysis using flow cytometry. PE-conjugated mouse anti-human CD19, CD34, CD45, HLA-DR, CD44, CD73, CD90 and CD105 antibodies were used. Mouse IgG1-PE was selected as an isotype control. All reagents were purchased from eBioscience (San Diego, CA, USA). The phenotype of hAd-MSCs is shown in Supplementary Figure S6.

### Adipogenic and osteogenic potentials of MSCs

Passage 3 hAd-MSCs cells from Donor #2 were seeded in a 6-well plate. When the cells reached 80% confluence, cells were cultured with adipogenic (Gibco, Grand Island, NY, USA) or osteogenic (Gibco) medium for 10 days. The conditioned media were replaced every 3 days, according to the manufacturer’s recommendations. Oil Red O (Solarbio, Beijing, China) was used to detect intracellular lipids, and Alizarin Red (Solarbio) was used to identify intracellular calcium (Supplementary Figure S6).

### MSC-CM preparation

Passage 3 hAd-MSCs were plated into 25-cm^2^ flasks at a density of 2 × 10^4^ cells/cm². When the cells reached 80% confluence, the complete medium was removed. Cells were then washed five times with 5 ml of ice-cold Dulbecco’s phosphate-buffered saline (DPBS) (Invitrogen Inc., Carlsbad, CA, USA). Then, 2.5, 5 or 7.5 ml of pre-warmed high-glucose, serum-free DMEM (Invitrogen Inc.) was added to one 25-cm^2^ flask, and the cells were cultured at 37 °C in a humidified atmosphere with 5% CO_2._ MSC-CM was collected separately at 6, 12, 24 and 48 h post-conditioning to determine the concentrations of human EGF, CXCL12 and Dkk-1.

### Cytokine array analysis of MSC-CM

The conditioned medium was generated using hAd-MSCs from Donor #1, Donor #2 and Donor #3, according to the above-mentioned procedures. Cytokine profiles were then assessed using the Proteome Profiler Human XL Cytokine Array Kit (R&D Systems, Minneapolis, MN, USA). The experimental procedures were conducted according to the manufacturer’s instructions.

### Concentrations of human EGF, CXCL12 and Dkk-1 in MSC-CM

In this study, the levels of cytokines, including human EGF, CXCL12 and Dkk-1, in MSC-CM (Donor #2) were tested using the Luminex assay. The kit was purchased from R&D Systems. The assay procedure was performed according to the manufacturer’s instructions. A quantitative analysis was conducted to identify the appropriate conditions for preparing MSC-CM. Under these conditions, MSC-CM was harvested, mixed and stored at −80°C until further use.

### Organoid preparation and culture

All the procedures used to obtain organoids were performed according to previously published methods^33^. Briefly, the small intestines of 6-week-old BALB/c mice (Vital River Laboratory Animal Technology Co., Ltd., Beijing, China) were freshly isolated and cut into small pieces (2–3 mm) after complete removal of the lumen contents. The tissue pieces were then incubated in 30 ml of DPBS (Invitrogen Inc.) containing 2 mM EDTA (Invitrogen Inc.) on ice with rotation for 30 min. Next, agitation was applied to release the crypt fragments. The cell suspension was passed through a 70-μm mesh (BD Biosciences, Franklin Lakes, NJ, USA). The filtrate was collected by centrifugation at 1000 r.p.m. for 5 min. Then, crypt fragments were added to ice-cold phenol-red-free, growth-factor-reduced Matrigel (BD Biosciences) at a ratio of 500 crypts per 50 μl of Matrigel. The Matrigel-containing crypts were placed in a 24-well plates, and 500 µl of IntestCult medium (Stemcell Technologies Inc., Canada) was added to each well. All cells were cultured at 37 °C with a humidified atmosphere of 5% CO_2_. All animals were anaesthetized before euthanasia, and all animal experimental procedures were approved by our local animal care and use committee.

### Epithelial organoid irradiation

A single dose of 10.5 Gy was administered to organoids using the X-RAD 320 Biological Irradiator (Stone Mountain, Atlanta, GA, USA). The following irradiation parameters were used: a dose rate of 1.5 Gy/min (300 kV, 11.9 mA) and an irradiation time of 7 min.

### In situ apoptosis in irradiated organoids

Organoids were cultured in a 24-well plate and then irradiated. Thereafter, the medium was removed from 8 wells of cultured organoids and replaced with 0.5 ml of MSC-CM (MSC-CM group, *n* = 8). The medium in another eight wells of irradiated organoids was not changed (IR-alone group, *n* = 8). The remaining eight wells of organoids that did not receive IR served as normal controls (Normal group, *n* = 8). Six hours later, the terminal deoxynucleotidyl transferase dUTP nick-end labelling (TUNEL) assay was performed to detect apoptotic cells within irradiated organoids. The In Situ Cell Death Detection Kit Fluorescein (Roche, Basel, Switzerland) was used to identify apoptotic cells within irradiated organoids. The medium used to culture organoids was discarded, and the wells were washed three times with 1 ml of pre-cooled PBS to detect apoptotic cells in a 24-well plate. Notably, the Matrigel should not be disturbed. Then, 0.5 ml of a 4% paraformaldehyde solution was added to each well, and the plates were incubated for 30 min at 4°C. Thereafter, the fixative solution was discarded, the wells were washed three times with 1 ml of pre-cooled PBS and the organoids were permeabilized with 0.1% sodium citrate containing 0.1% Triton X-100 (Sigma-Aldrich, St. Louis, MO, USA) for 5 min. The wells were then washed three times with 1 ml of pre-cooled PBS. Next, 1 ml of pre-cooled PBS was added to each well, and the Matrigel in each well was disrupted with a 1-ml pipette. The PBS containing dissociated organoids was transferred to a 1.5-ml tube, and the tube was centrifuged at 1000 × *g* for 10 min. Next, the PBS was discarded, and 50 μl of TUNEL reaction mixture were added to each tube. The organoids were incubated at 37 °C in a humidified atmosphere in the dark for 1 h. Thereafter, the organoids were washed three times with 1 ml of pre-cooled PBS. Finally, 200 μl of PBS containing 1× 4',6-diamidino-2-phenylindole (DAPI) (ThermoFisher Scientific, Waltham, MA, USA) was added to each well to counterstain the nuclei. Confocal images were captured at excitation wavelengths of 488 nm (for dUTP-FITC) and 405 nm (for DAPI) using a Leica SP5 microscope (Germany).

### Establishment of MSCs over-expressing the mouse *cxcl12* gene

The lentivirus transfection system was used to establish MSCs over-expressing the mouse *cxcl12* gene. Briefly, information for the mouse *cxcl12* gene was retrieved from NCBI (www.ncbi.nlm.nih.gov) and transcript variant 1 (NM_021704.3) encoded the mouse CXCL12 protein. The corresponding coding sequence (CDS) was then determined, and the following primers were designed to amplify the *cxcl12* CDS: sense, 5′-CGCGGATCCATGGACGCCAAGGTCGTCGCC-3′ and anti-sense: 5′-TTGGCGCGCCTTACTTGTTTAAAGCTTTCTCCAGG-3′ (product size: 270 bp). Next, the *cxcl12* gene was amplified from mouse tail genomic DNA by PCR. Using T4 ligase (Takara Bio Inc., Shiga, Japan), the PCR products of the *cxcl12* gene were linked to the *Amp* resistance gene in a lentivirus plasmid (Supplemental Figure S3) that had been digested with *Asc*I and *Bam*HI (Takara Bio Inc.) at a locus downstream of the *Ef1a* promoter. The empty vector *Lv-Ef1a-IRES-eGFP* served as a control. After amplifying the established plasmids in *E. coli*, the plasmids were sequenced to determine whether they contained the correct *cxcl12* CDS, namely, *Lv-Ef1a-cxcl12-IRES-eGFP*. Next, HEK293T cells were transfected with an established vector plasmid plus pVSVG and delta 8.91 using the Fugene HD Transfection Reagent (Roche) supplied with the package plasmids. After 48 h, the supernatants were collected to determine the virus titre using fluorescence-activated cell sorting analysis. Finally, passage 3 MSCs were infected with the packaged virus. GFP-positive cells were sorted and cultured at 37 °C in a humidified atmosphere with 5% CO_2_. Detailed procedures for expanding transfected MSCs were the same as those described above.

### Western blotting

Total proteins were prepared from organoids using RIPA lysis buffer (Sigma-Aldrich) plus 1 × Protease Inhibitor Cocktail (Sigma-Aldrich), 1 × Phosphatase Inhibitor Cocktail 2 (Sigma-Aldrich) and 1 × Phosphatase Inhibitor Cocktail 3 (Sigma-Aldrich). Heat-denatured proteins were used for the western blotting experiments. Sodium dodecyl sulphate-polyacrylamide gel electrophoresis was performed, and then the proteins were hybridized to a PVDF membrane (Millipore, MN, USA). The membrane was incubated in TBS-T (Tris-buffered saline with Tween-20) buffer containing 5% of bovine serum albumin (BSA) for 2 h at room temperature. Thereafter, the PVDF membrane was incubated with shaking in primary antibody solution overnight at 4 °C. On the following day, the membrane was washed three times with TBS-T buffer for 10 min each. Thereafter, the membrane was incubated in secondary antibody solution with shaking for 2 h at room temperature. After washing the membrane using TBS-T, a chemiluminescent ECL reagent was used for the development step (Millipore). All antibodies were diluted at a concentration recommended by the manufacturers. The antibodies are listed in the Supplementary Table (see online Supplementary Information).

### Blocking assay

To test the specificity of the CXCL12–CXCR4 axis for the phosphorlation of Akt at Ser473 and Erk1/2 at Thr202/Thr204, 1 μg/ml of CXCL12 neutralizing antibody (Abcam), 10 μM of AMD3100 (Sigma-Aldrich), 10 μM of LY294002 (Sigma-Aldrich) and 10 μM of U0126 (Cell Signalling Technology, MA, USA) were separately added to the Lv-*cxcl12*-MSC-CM to block mouse CXCL12 in CM, CXCR4 on organoid cells, PI3K and MEK. CXCL12 neutralizing antibodies, AMD3100, LY294002 and U0126, were used at the concentrations recommended by the manufacturers. Six hours later, total proteins of irradiated organoids were harvested for western blotting.

### Cell viability analyses

In this assay, 10 μl of Matrigel containing approximately 10 organoids was placed in a 96-well plate. One hundred microlitres of organoid growth medium was added to each well, and 24 h later, 40 wells of the organoids were irradiated using a single fraction of 10.5 Gy. Eight wells of the organoids were used as the healthy control. In the irradiated wells, the medium from each of eight wells was replaced with Lv-MSC-CM, Lv-*cxcl12*-MSC-CM and Lv-*cxcl12*-MSC-CM containing 10 μM of AMD3100 and Lv-*cxcl12*-MSC-CM containing 10 μM of AMD3100 of U0126. The remaining eight wells were IR-alone controls. Six hours later, the organoids were dissociated into a single-cell suspension using Gentle-Cell Dissociation Reagent (Stemcell Technologies Inc.) because this reagent is enzyme-free. Next, 20,000 cells from each sample were centrifuged at 1,000 × *g* for 5 min, and the supernatant was discarded. The cells were stained using 1 × PrestoBlue Cell Viability Reagent (ThermoFisher Scientific) for 10 min, and the absorbance was determined using a BioTek stripe reader (Winooski, VT, USA) at an excitation wavelength of 570 nm. The wells containing only 1× PrestoBlue solution were set as blank controls.

### Immunocytochemical staining

Immunocytochemical (ICC) staining for phosphorylated Erk1/2 at Thr202/Thr204 was performed 6 h post IR in cells grown in a 24-well plate. Six wells of irradiated organoids received Lv-MSC-CM treatment (Lv-MSC-CM group; *n* = 6), six wells of irradiated organoids received Lv-*cxcl12*-MSC-CM treatment (Lv-*cxcl12*-MSC-CM group; *n* = 6), six wells of irradiated organoids served as experimental controls (IR-alone group; *n* = 6), and six wells of unirradiated organoids served as healthy controls (Normal group; *n* = 6). As mentioned above, normal or irradiated organoids were fixed and ruptured sequentially. The Matrigel was then disturbed to release the organoids, and the organoids were immersed in PBS containing 10% FBS (v/v) and 5% BSA (w/v) for 30 min to block endogenous non-specific antigens. Thereafter, primary antibody was added and incubated with the organoids overnight at 4°C with rotation. Subsequently, the organoids were washed three times with 1 ml of 1× PBS-Tween (PBS-T) buffer. Next, FITC-conjugated secondary antibody was incubated with the organoids for 1 h at 37 °C. After washing, the nuclei were counterstained with DAPI (ThermoFisher Scientific). The primary antibody was rabbit anti-mouse phosph-Erk1/2 Thr202/Thr204 antibody, and the secondary antibody was FITC-conjugated goat anti-rabbit IgG (H + L). The working concentrations of the antibodies were generated according to the manufacturer’s instructions. The antibodies are listed in the Supplementary Table (see online Supplementary Information**)**.

### Real-time PCR

In 24-well plates, six wells of irradiated organoids were subjected to Lv-MSC-CM treatment (Lv-MSC-CM group; *n* = 6), six wells of irradiated organoids were exposed to Lv-*cxcl12*-MSC-CM treatment (Lv-*cxcl12*-MSC-CM group; *n* = 6), six wells of irradiated organoids served as experimental controls (IR-alone group; *n *= 6) and six wells of normal organoids served as healthy controls (Normal group; *n* = 6). After 6 h, total RNA was extracted using TRIzol reagent (Invitrogen Inc.), and 1 μg of total RNA was used to synthesize first-strand cDNAs using the RT-PCR Kit (Takara Bio Inc.). Total first-strand cDNAs were used in a microsystem together with the primers, SYBR Green I (Roche) oligonucleotide probe, nucleotides and Taq DNA polymerase (Takara Bio Inc.). The PCR assay was then performed using a Bio-Rad instrument for 45 cycles. The primer sequences were as follows:

*PCNA*: (forward) 5′-AGAGCTTGGCAATGGGAACA-3′, (reverse) 5′-CTCTACAACAAGGGGCACATC-3′. The production size was 196 bp.

*CXCR7*: (forward) 5′-GTGTCCCACCATGCCTAACA-3′, (reverse) 5′-TGCAGGCGAGGAAGAAGATG-3′. Relative production size was 314 bp. *GAPDH*: (forward) 5′-GAAGGTGACAGCAGTCGGTT-3′, (reverse) 5′-GGGACTTCCTGTAACAACGCAT-3′. The product size was 218 bp.

### RIII model

One hundred and fifty 6-week-old male BALB/c nude mice weighing 16 ± 0.5 g were purchased from Vital River Laboratory Animal Technology Co., Ltd. (Beijing, China). The mice were intraperitoneally anaesthetized with 100 μl of 10% chloral hydrate. A single dose of 10.5 Gy was administered to the abdomen using the X-RAD 320 Biological Irradiator (Stone Mountain). The following parameters were used for abdominal irradiation: an irradiation field of 1.5 cm × 1.5 cm in the central zone of the abdomen and a dose rate of 1.5 Gy/min (300 kV, 11.9 mA) for 7 min.

### Animal study designation

After irradiation, the mice were intraperitoneally injected with 5 × 10^5^ Lv-MSCs or Lv-*cxcl12*-MSCs, namely, the Lv-MSC group and the Lv-*cxcl12*-MSC group, and normal mice and irradiated mice, respectively, served as healthy controls (Normal group) and experimental controls (IR-alone group). Each group contained 18 mice. First, the mice were housed for 30 days to analyse their survival. Second, individual mice were euthanized on days 1, 3 and 7 post IR to harvest the small intestine at a site 1.5-cm distal from the ileocecal junction for histological and serum cytokine analyses. The number of mice used for the histological and cytokine analyses is indicated in the corresponding figures. All animals were anaesthetized before euthanasia, and all animal experimental procedures were approved by our local animal care and use committee.

### In vivo distribution of Lv-cxcl12-MSCs

Nine BALB/c nude mice were used to trace the Lv-*cxcl12*-MSCs after peritoneal injection. Herein, three normal mice without Lv-*cxcl12*-MSCs treatment were used to establish the background fluorescence. Three normal mice treated with Lv-*cxcl12*-MSCs were used to identify the specific locations of Lv-*cxcl12*-MSCs in comparison to the remaining three irradiated mice, which received Lv-*cxcl12*-MSCs treatment. In this experiment, GFP fluorescence signals were detected using a small animal imaging system (Pearl Trilogy, NE, USA). The images were captured on days 1, 3 and 7 after injection of Lv-*cxcl12*-MSCs. Red fields representing enrichment of Lv-*cxcl12*-MSCs in the abdomen and chest of mice were separately analysed using Image Pro Plus 6.0 software (Media Cybernetics, Rockville, MD, USA).

### Lv-*cxcl12*-MSCs in irradiated small intestine

Nine irradiated BALB/c nude mice received an intraperitoneal injection of Lv-*cxcl12*-MSCs. Each mouse was injected with 5 × 10^5^ cells. Each set of three mice was euthanized 1, 3 and 7 days post IR, and total proteins were harvested from 5 mg of fresh intestinal tissue using the procedures described in the western blotting section. Rabbit anti-GFP antibody was used to detect Lv-*cxcl12*-MSCs in irradiated small intestine using the western blotting assay. The grey density of each sample was analysed using Image Pro Plus 6.0 Software (Media Cybernetics).

### Histological analyses

Paraffin-embedded 4-μm-thick sections were used for haematoxylin and eosin (H&E), IHC and TUNEL assays. Briefly, the sections were dewaxed and rehydrated. For H&E staining, the sections were immersed in haematoxylin (Sigma-Aldrich) for 5 min. Thereafter, they were washed with running water and then differentiated in 1% hydrochloric ethanol for 30 s. Next, they were immersed in running water to enable the nuclei to return to a blue colour. The sections were then stained with eosin for 5 min and washed with running water. Finally, they were dehydrated using a gradient of alcohol solutions and immersed in xylene to induce tissue transparency.

For IHC staining, endogenous peroxides were blocked. Thereafter, antigen retrieval was performed in 1 × sodium citrate buffer (Abcam, Cambridge, MA, USA). After blocking endogenous non-specific antigens using PBS containing 10% FBS (v/v) and 5% BSA (w/v) for 30 min, the sections were incubated with primary antibodies overnight at 4 °C. After incubation, the sections were washed with 1 × PBS-T buffer and then incubated with secondary antibodies for 1 h at 37°C. Haematoxylin was used for nuclear counterstaining. Primary antibodies included rabbit anti-GFP, rabbit anti-mouse PCNA, rabbit anti-mouse phospho-Erk1/2 Thr202/Thr204, rabbit anti-mouse phospho-Akt Ser473, rabbit anti-mouse CXCR4 and rabbit anti-mouse Lgr5 (GPCR49). Working concentrations of the primary antibodies were generated according to the manufacturer's instructions. The rabbit-specific HRP/DAB (ABC) Detection IHC Kit (Abcam) was used to detect positive cells.

For the TUNEL assay, the sections were dewaxed, rehydrated and subjected to antigen retrieval procedures as described above. Next, 0.1% sodium citrate containing 0.1% Triton X-100 was used to treat the sections for 5 min. Thereafter, after discarding the permeabilizing solution and washing the sections using PBS, each section was treated with 50 μl of TUNEL reaction mixture for 2 h at 37 °C. The sections were then washed three times with pre-chilled PBS for 5 min each. Next, 50 μl of converter-peroxidase (POD) was added to each section and incubated for 30 min at 37 °C. Finally, 50 μl of DAB (Abcam) was used to identify apoptotic cells. The procedures were performed according to the instructions provided with the In Situ Cell Death Detection Kit, POD (Roche). Nuclei were counterstained using haematoxylin.

### Cytokine array of serum samples

At 3 days post IR, serum samples were collected from all groups. Each group consisted of ten independent samples. Fifty microlitres of each sample was mixed with other samples from the same group and brought to a total volume of 500 μl, which was then used for the cytokine array analysis. The Proteome Profiler Mouse XL Cytokine Array Kit (R&D Systems) was used to screen for differences in the indicated cytokines among the groups. All experimental procedures were performed according to the manufacturer’s instructions.

### ELISA

At 3 days post IR, serum samples were collected from all groups. Each group contained ten independent samples. The Mouse CXCL12/SDF-1alpha Quantikine ELISA Kit (R&D Systems), Mouse IL-1alpha ELISA Kit (Invitrogen Inc.) and Mouse IL-6 ELISA Kit (Invitrogen Inc.) were used to measure the serum concentrations of mouse CXCL12, IL-1α and IL-6, respectively. In addition, the Mouse CXCL12/SDF-1alpha Quantikine ELISA Kit (R&D Systems) was used to measure mouse CXCL12 concentrations in Lv-*cxcl12*-MSC-CM. All experimental procedures were performed according to the manufacturer's instructions. The cytokine concentrations were assessed using a BioTek stripe reader (Winooski, VT, USA) at a wavelength of 450 nm with 630-nm wavelength correction.

### Statistical analysis

All data were analysed using GraphPad Prism 5 software (GraphPad software Inc., La Jolla, CA, USA). The data are presented as the mean ± standard deviation (S.D.).The Kaplan–Meier method was used to analyse mouse survival. Unpaired *t* tests were used to determine the significance of differences between two groups, and one-way analysis of variance (ANOVA) was used to determine the significance of differences among three or more groups. A *P* value ≤0.05 represented a significant difference.

## Electronic supplementary material

Supplemental information
