# Supplementary material for: Mesenchymal stem cells over-expressing cxcl12 enhance the radioresistance of the small intestine
Source: Cell Death Dis. 2018 Feb 5;9(2):154. doi: 10.1038/s41419-017-0222-1 (PMC5833479; doi:10.1038/s41419-017-0222-1)
Supplement: Supplementary file 1 — Supplemental information [file 41419_2017_222_MOESM1_ESM.docx]

**Title: Mesenchymal stem cells over-expressing *cxcl12* enhance the radioresistance of the small intestine**

Author list: Pengyu Chang , Boyin Zhang, Lihong Shao, Wei Song, Weiyan Shi, Libo Wang, Tiankai Xu, Dong Li , Xiuzhu Gao, Yaqin Qu, Lihua Dong^*^ and Jin Wang^*^

**Supplemental Methods and Materials**

**Flow-cytometric analysis for cell apoptosis within irradiated organoids.**

In a 24-well plate, 6 wells of organoids were not irradiated. They were set as healthy controls. 12 wells of organoids were irradiated using a single fraction of 10.5 Gy. Thereafter, the medium in 6 wells were changed into 500 μl of MSC-CM. The rest 6 wells of irradiated organoids were belonging to IR alone group. 6 hours later, the organoids were dissociated into single cells using Gentle-Cell Dissociation Reagent (STEMCELL Technologies Inc.) according to manufacture’s instruction. Then, the Apoptosis Detection Kit (MULTI SCIENCES, Hangzhou, China) was used. The experiment was performed according to the manufacturer’s instruction. Herein, cells, double-positive for Annexin-V and Propidium iodide (PI), were defined as cells at late apoptotic stage. And cells, single-positive for Annexin-V, were regarded as cells at early apoptotic stage. Cells at early apoptotic stage or at late apoptotic stage were gated for comparing among groups.


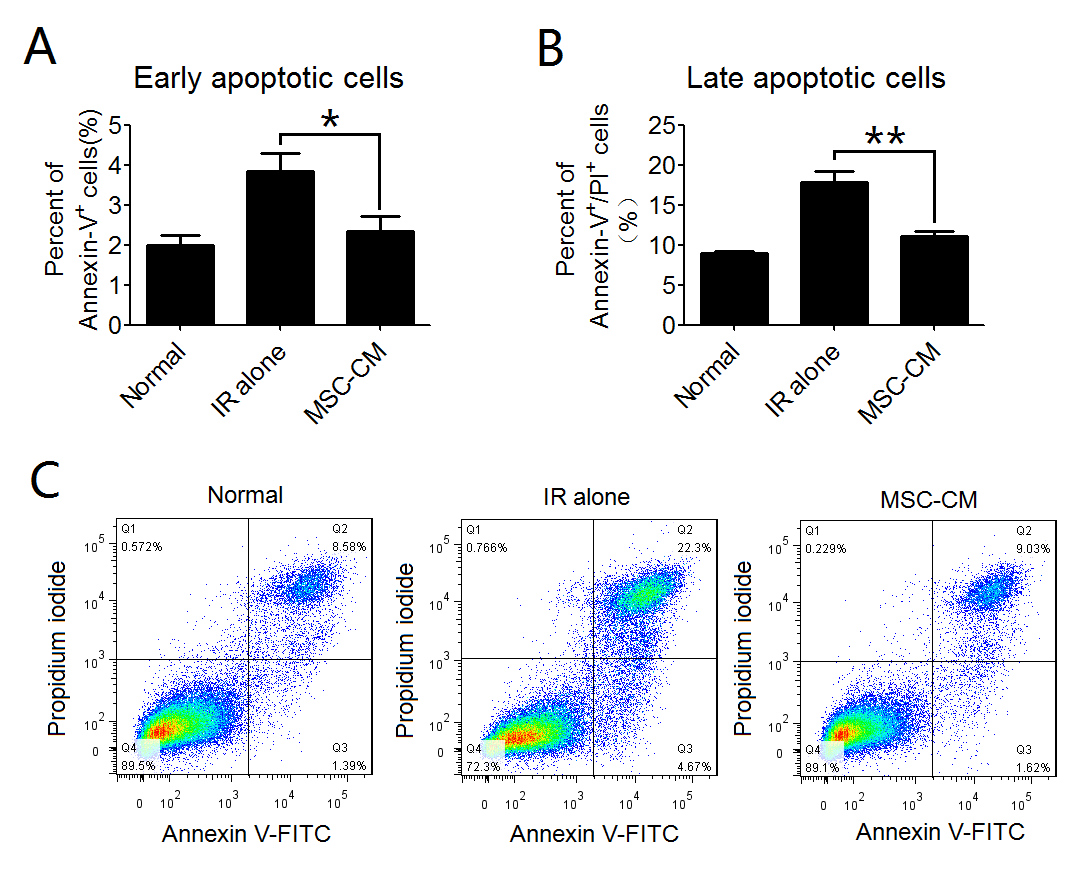


**Supplemental Figure S1**

**Flow-cytometric analysis for cell apoptosis within irradiated organoids using Annexin-V/Propidium iodide (PI) method.**

(A) Comparing the amounts of cells at early apoptotic stage (Annexin-V+) within irradiated organoids. Each group had 6 independent measurements (n = 6). Data represented Mean ± SD. * *P* < 0.05: Significantly high (IR alone group versus MSC-CM group by Unpaired *t* test).

(B) Comparing the amounts of cells at late apoptotic stage (Annexin-V+/PI+) within irradiated organoids. Each group had 6 independent measurements (n = 6). Data represented Mean ± SD. ** *P* < 0.01: Significantly high (IR alone group versus MSC-CM group by Unpaired *t* test).

(C) Flow-cytometric scatterings for Annexin-V-FITC and PI among groups. Up-right quadrant: Annexin-V+/PI+ cells at late apoptotic stage; Bottom-right quadrant: Annexin-V+ cells at early apoptotic stage. The number in each quadrant represented the cell ratio


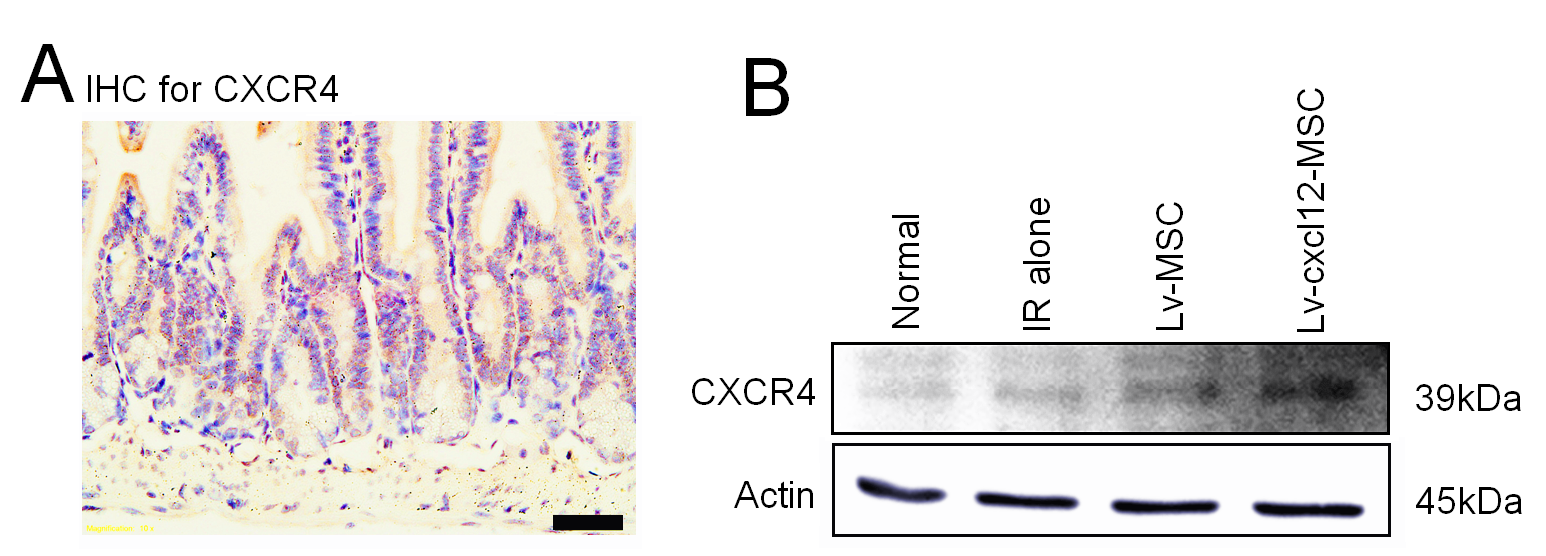


**Supplemental Figure S2**

**Distribution of CXCR4-positive cells *in vivo*.**

1. IHC-staining for mouse CXCR4 on small intestinal section. Magnification at 400 ×; Bar: 50 μm.
2. Western blotting for mouse CXCR4 by organoid cells at 6 hours post-IR. β-Actin was used as internal control.


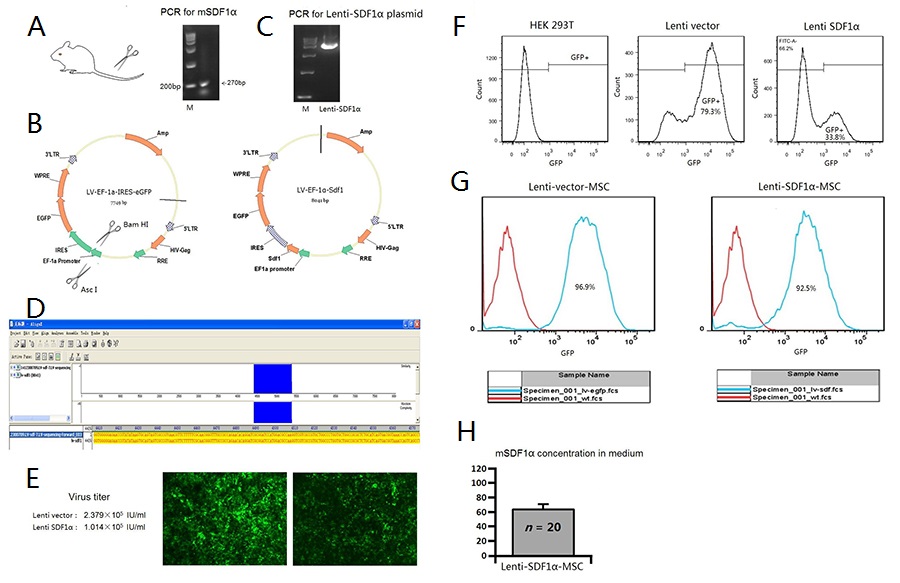


**Supplemental Figure S3**

**Constructing hAd-MSCs over-expressing mouse *cxcl12* gene.**

1. PCR amplifying mouse *cxcl12* CDS cDNA ;
2. Inserting mouse *cxcl12* into Lenti-plasmid profile;
3. PCR amplifying for Lenti-*cxcl12* plasmid;
4. Sequencing for Lenti-*cxcl12* plasimid;
5. Packaging Lenti-*cxcl12* plasmid in HEK 293 T cells;
6. FACS for determining transfection efficacy of Lenti-*cxcl12* plasmid;
7. Transfecting packaged Lenti-*cxcl12* into hAd-MSCs;
8. ELISA for detecting mouse CXCL12 concentration in Lv-*cxcl12*-MSC-CM. Twenty independent sample were measured (n = 20). Data was shown as mean ± S.D.


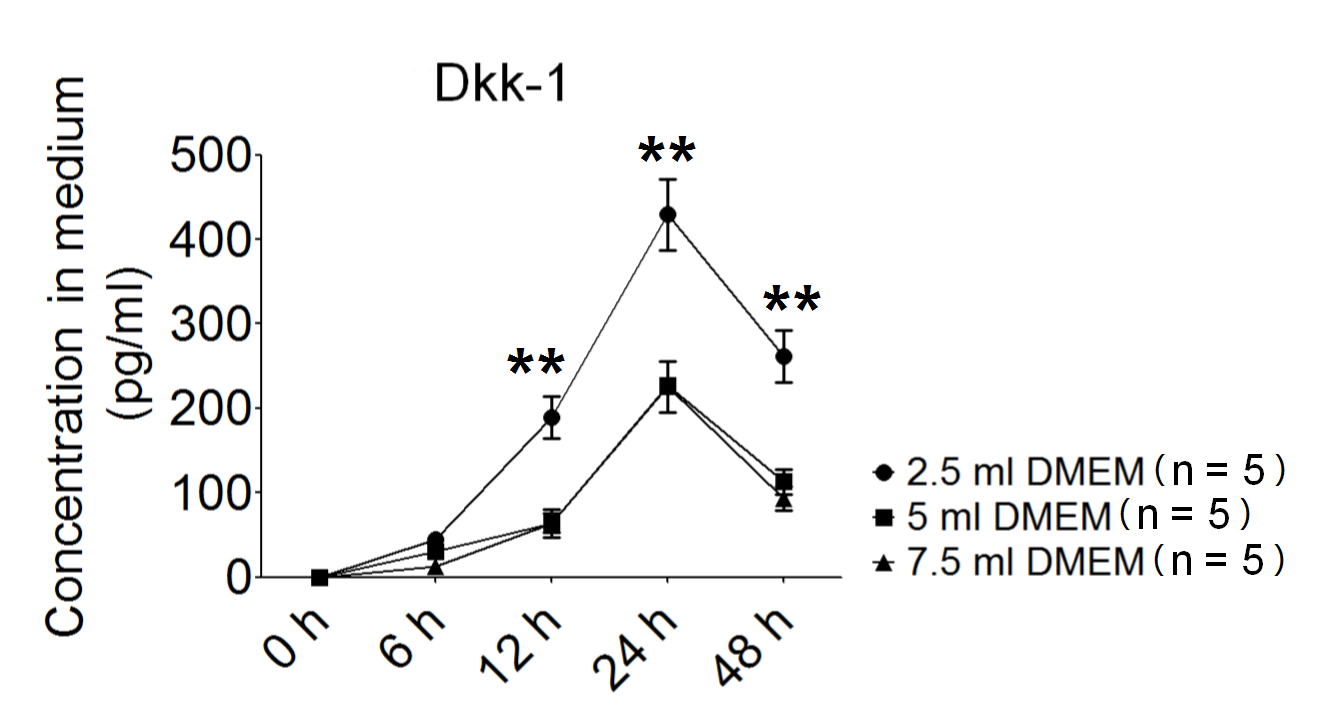


**Supplemental Figure S4**

**Human Dkk-1 levels in MSC-CM.**

Each time point had five independent mesurments (n = 5). Data represented mean ± S.D. ***P* ≤ 0.001: significantly higher (2.5 ml of DMEM group versus other two groups, One-way ANOVA analysis).


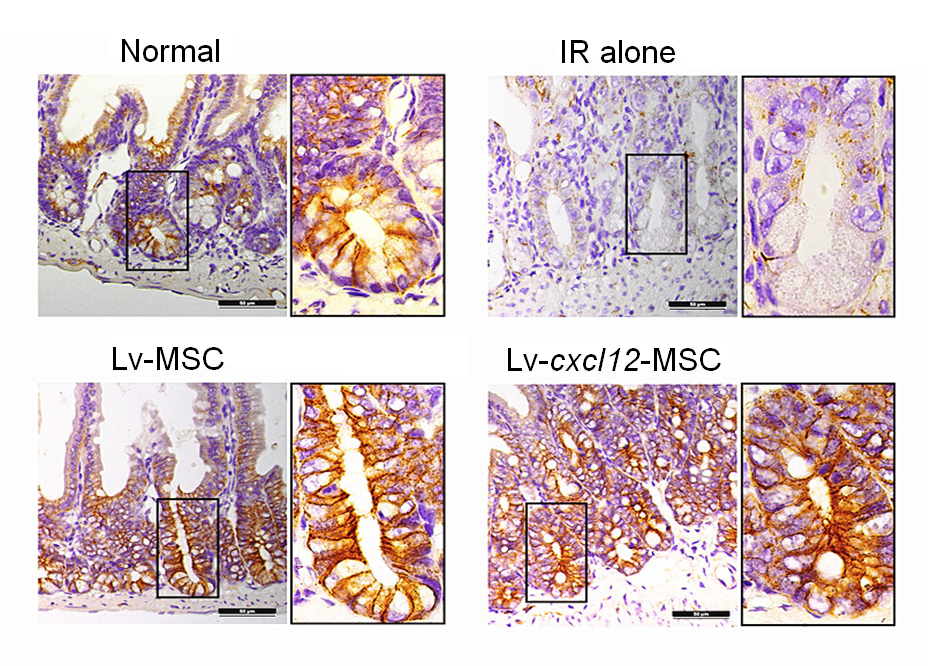


**Supplemental Figure S5**

**Distribution of active β-catenin^+^ cells within irradiated intestinal epithelium.**

IHC staining for active β-catenin was performed at 3 days post-IR. Representative

images were captured for all groups. Magnification at 400×; scale bar: 50 μm. The

images in black frames were at 1000× magnification.


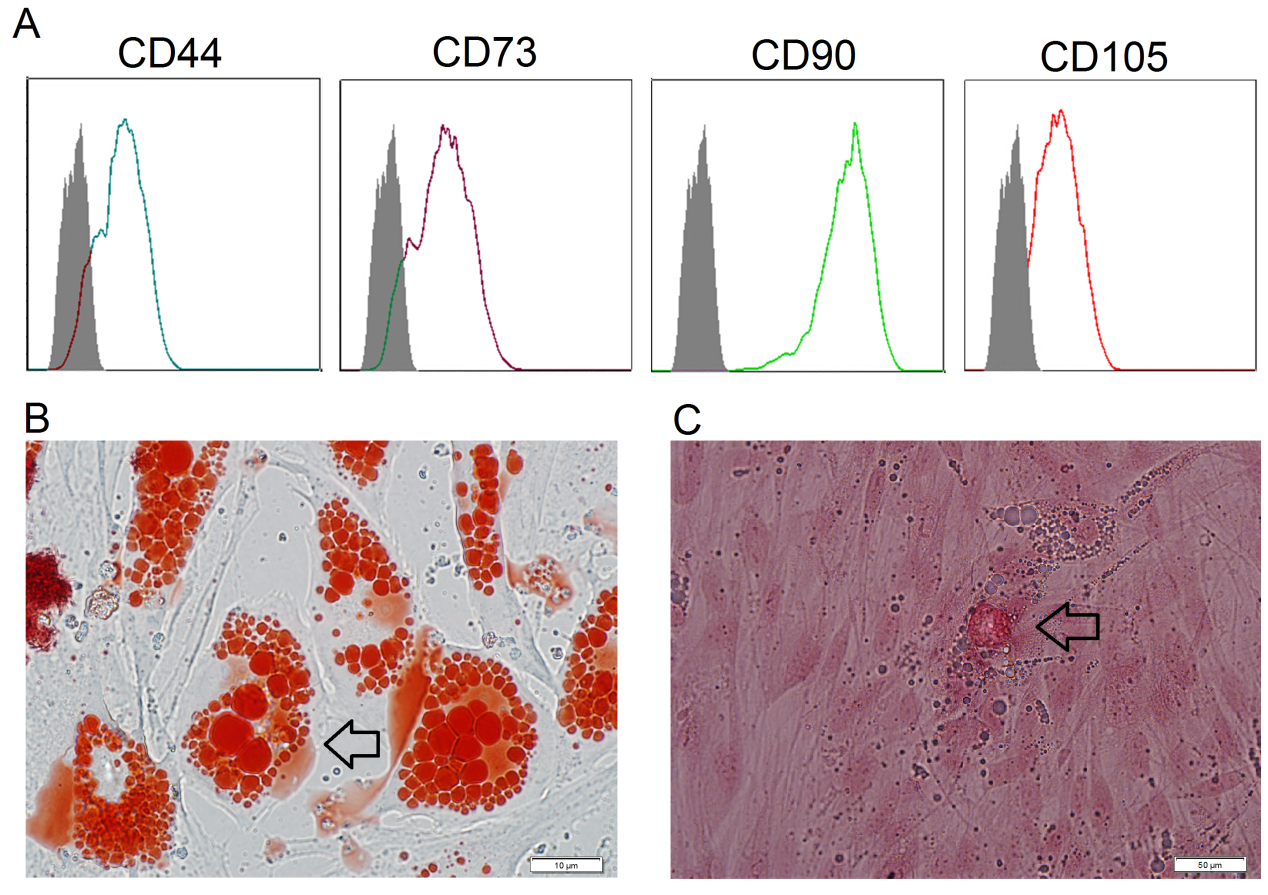


**Supplemental Figure S6**

**Identification of hAd-MSCs.**

The hAd-MSCs were from Donor #2. A: FACS for cell phenotype, including CD44, CD73, CD90 and CD105; B: Adipogenic potential of hAd-MSCs. Black arrow: fat lipid. Magnification at 1000 ×; Scale bar: 10 μm. C: Osteogenic potential of hAd-MSCs. Black arrow: calcium. Magnification at 1000 ×; Scale bar: 10 μm.

**Supplemental Table** Antibody information

| Primary/Sencondary | Antibody name (Cat. number) | Company |
| --- | --- | --- |
| Primary | Rabbit anti-mouse Akt (#4691) | Cell Signaling Technology |
| Primary | Rabbit anti-mouse phospho-Akt Ser 473 (#4060) | Cell Signaling Technology |
| Primary | Rabbit anti-mouse caspase3 (#9662) | Cell Signaling Technology |
| Primary | Rabbit anti-mouse cleaved caspase3 (#9664) | Cell Signaling Technology |
| Primary | Rabbit anti-mouse PUMA (#24633) | Cell Signaling Technology |
| Primary | Rabbit anti-mouse actin (#4970) | Cell Signaling Technology |
| Primary | Rabbit anti-mouse Erk1/2 (#4695) | Cell Signaling Technology |
| Primary | Rabbit anti-mouse phospho-Erk1/2 Thr202/Thr204 (#4370) | Cell Signaling Technology |
| Primary | Rabbit anti-mouse PCNA (#13110) | Cell Signaling Technology |
| Primary | Rabbit anti-mouse histone 3 (#4499) | Cell Signaling Technology |
| Primary | Rabbit anti-mouse phospho-histone 3 Ser10 (#53348) | Cell Signaling Technology |
| Primary | Rabbit anti-mouse CK1 (ab82426) | Abcam |
| Primary | Rabbit anti-mouse GSK3β (#12456) | Cell Signaling Technology |
| Primary | Rabbit anti-mouse phospho-GSK3β Ser9 (#9322) | Cell Signaling Technology |
| Primary | Rabbit anti-mouse β-catenin (#8480) | Cell Signaling Technology |
| Primary | Rabbit anti-mouse non-phospho-β-catenin Ser33/37/Thr41 (#8814) | Cell Signaling Technology |
| Primary | Rat anti-mouse CD44 variant 6 (NB100-64818) | Novus Biologicals  CO, USA |
| Primary | Rabbit anti-mouse CXCR4 (ab124824) | Abcam  MA, USA |
| Primary | Rabbit anti-mouse GPCR RDC1 (ab38089) | Abcam |
| Primary | Rabbit anti-GFP antibody(#2956) | Cell Signaling Technology |
| Secondary | Goat anti-rabbit IgG (H+L)-HRP (ab6721) | Abcam |
| Secondary | Goat anti-rat IgG (H+L)-HRP (ab97057) | Abcam |
| Primary | Rabbit anti-CXCL12 antibody (ab9797) | Abcam |
| Secondary | Goat anti-rabbit IgG (H+L)-FITC (ab6717) | Abcam |
| Primary | Rabbit anti-mouse GPCR GPR49 (ab75732) | Abcam |
